# Biofouling of Water Treatment Membranes: A Review of the Underlying Causes, Monitoring Techniques and Control Measures

**DOI:** 10.3390/membranes2040804

**Published:** 2012-11-21

**Authors:** Thang Nguyen, Felicity A. Roddick, Linhua Fan

**Affiliations:** School of Civil, Environmental and Chemical Engineering, Water: Effective Technologies and Tools (WETT) Centre, RMIT University, Melbourne, VIC. 3001, Australia; Email: thang.nguyen@rmit.edu.au (T.N.); linhua.fan@rmit.edu.au (L.F.)

**Keywords:** membrane biofouling, biofilm, extracellular polymeric substances, biofouling control, biofouling monitoring

## Abstract

Biofouling is a critical issue in membrane water and wastewater treatment as it greatly compromises the efficiency of the treatment processes. It is difficult to control, and significant economic resources have been dedicated to the development of effective biofouling monitoring and control strategies. This paper highlights the underlying causes of membrane biofouling and provides a review on recent developments of potential monitoring and control methods in water and wastewater treatment with the aim of identifying the remaining issues and challenges in this area.

## 1. Introduction

Membrane fouling is a major problem encountered in membrane filtration processes, and it is a major factor in determining their practical application in water and wastewater treatment and desalination in terms of technology and economics. Membrane fouling includes inorganic fouling/scaling, organic fouling, particulate/colloidal fouling and biofouling (or microbial/biological fouling). Fouling due to organic and inorganic components and microorganisms can occur simultaneously, and these components may interact in terms of mechanism [1]. Biofouling represents the “Achilles heel” of the membrane process because microorganisms can multiply over time; even if 99.9% of them are removed, there are still enough cells remaining which can continue to grow at the expense of biodegradable substances in the feed water [2]. Biofouling can be considered as a biotic form of organic fouling, while fouling caused by organic matter derived from microbial cellular debris can be considered as an abiotic form of biofouling [3]. Biofouling has been known as a contributing factor to more than 45% of all membrane fouling [4] and has been reported as a major problem in nanofiltration (NF) and reverse osmosis (RO) membrane filtration [5]. The focus of this review is therefore on biofouling in NF and RO systems.

Biofouling can have several adverse effects on membrane systems [6,7,8,9,10,11] such as:

- Membrane flux decline due to the formation of a low permeability biofilm on the membrane surface.
- Increased differential pressure and feed pressure being needed to maintain the same production rate due to biofilm resistance.
- Membrane biodegradation caused by acidic by-products which are concentrated at the membrane surface. For example, cellulose acetate membrane has been found to be more susceptible to being biodegraded.
- Increased salt passage through membrane and reduced quality of the product water due to the accumulation of dissolved ions in the biofilm at the membrane surface thus increasing the degree of concentration polarization.
- Increased energy consumption due to higher pressure being required to overcome the biofilm resistance and the flux decline.

Several methods and strategies for membrane biofouling control have been developed over the last two decades. This paper provides a review of the role of extracellular polymeric substances (EPS) in membrane biofouling and recent advancements in the development of methods for monitoring and controlling biofouling. The remaining issues and challenges in biofouling research are discussed, and areas for further work are recommended.

## 2. Biofilm Formation and Characterization Techniques

### 2.1. Transport of Microorganisms to the Membrane Surface

The forces which transport microorganisms to a surface have been studied by Marshall and Blainey [12]. Fluid dynamic forces are the major mechanism for transporting microorganisms to the membrane surface. In spiral wound RO, the spacer between the membrane envelopes is designed to promote turbulence and assist in transporting the feed water back to the bulk stream. However, in creating turbulence, areas with low flow are developed just downstream from each crossmember in the spacer and thus fouling can build up in these areas [13]. Matter that is caught in the spacer is trapped until the flow pattern changes. During this time other forces are in operation. For example, Brownian motion will assist in transporting non-motile cells to the vicinity of the membrane surface and motile cells which exhibit chemotaxis will advance to the membrane surface where nutrients are concentrated [14].

### 2.2. Microbial Attachment and Biofilm Formation on Membrane Surfaces

The attachment of microbial cells to the membrane surface is the first step of membrane biofouling [15], leading to the formation of the biofilm layer. The biofilm may comprise populations of different types of microorganisms (e.g., bacteria, algae, protozoa and fungi). Initial microbial attachment is mediated by electrokinetic and hydrophobic interactions [16], and is generally followed by cell growth and multiplication at the expense of soluble nutrients in the feed water or adsorbed organics on the membrane surface [17]. The extracellular polymeric substances (EPS) excreted by microorganisms anchor the cells to the substratum and further condition and stimulate additional microbial colonization of the membrane surface [18]. 

The attachment of microorganisms to the membrane surface is affected by factors such as membrane material (substratum nature) [9], the roughness of the membrane surface [19], hydrophobicity [20] and membrane surface charge [20]. Mc Eldowney and Fletcher [21] showed that microbial adhesion increased with increasing electrolyte concentration; however, other authors [22,23] demonstrated that there was no significant correlation between initial adhesion and electrolyte concentration. Ridgway *et al.* [22] found that pH has little effect on bacterial adhesion to cellulose acetate membranes. Similarly, Sadr Ghayeni *et al.* [24] found that initial adhesion of three sewage bacteria belonging to the genus *Pseudomonas* to RO and NF membranes was not affected by pH in the range 4–8, but was influenced by the ionic strength of the feed solution. A low ionic strength milieu would reduce the bacterial attachment. Permeation drag, back-diffusion transport and cross-flow velocity were also found to influence the attachment of bacteria and their growth on the membrane surface [25]. Factors affecting the adhesion of microorganisms to membrane surfaces are summarized in Table 1.

**Table 1:** Factors affecting microorganism adhesion to membrane surfaces [14].

Microorganism	Surface	Feed water
Species	Chemical composition	Temperature
Composition of mixed population	Surface charge	pH
Population density	Surface tension	Dissolved organic matter
Growth phase	Hydrophobicity	Dissolved inorganics
Nutrient status	Conditioning film	Suspended matter
Hydrophobicity	Roughness	Viscosity
Charges	Porosity	Shear forces
Physiological responses	–	Boundary layer
–	–	Flux

The sequence of biofilm formation includes (a) the adsorption of organic species and suspended particles on the wetted membrane surface to form a conditioning film; (b) the transport of the microbial cells to the conditioning film; (c) the attachment of the microbial cells to the membrane surface; (d) the growth and metabolism of the attached microorganisms and biofilm development; (e) the limitation of biofilm growth by fluid shear forces (detachment process) to achieve a steady state fouling resistance [26]. 

The microorganisms in a biofilm live in a matrix of hydrated extracellular polymeric substances that form their immediate environment. In most biofilms, the microorganisms account for less than 10% of the dry mass whereas the matrix of extracellular materials, which is mostly produced by the organisms themselves, can account for over 90% [27]. Under most environmental conditions, organic carbon compounds provide the nutrients for growth and energy supply to the biomass. A wide range of organic compounds such as carboxylic acids, amino acids, proteins and carbohydrates promote bacterial growth in the aquatic environment and biofilm growth causing operational problems such as clogging of the feed channel at concentrations as low as micrograms per liter [28].

### 2.3. The Role of Extracellular Polymeric Substances in Membrane Biofouling

Extracellular polymeric substances (EPS) are mainly high molecular weight secretions of microorganisms and consist of various organic substances such as polysaccharides, proteins, nucleic acids and lipids [29]. EPS bind the microbes together in a three-dimensional matrix and so affect the physico-chemical characteristics of the microbial aggregates such as mass transfer, surface characteristics, adsorption ability and stability [30]. The EPS are distributed in layers of varying depth through the biofilm [31]. They establish the structural and functional integrity of microbial biofilms, and significantly contribute to the organization of the biofilm community [32]. They also contribute to the mechanical stability of the biofilms, enabling them to withstand considerable shear forces [33]. 

EPS produced at the solid surface promote microbial adhesion by altering the physicochemical characteristics of the colonized surface such as charge, hydrophobicity, and roughness [34]. They create scaffolds with suitable physical characteristics and interconnected pore structures that promote cell attachment [35]. Cell adhesion to solid surfaces is inhibited by electrostatic interaction at low EPS concentration but enhanced by polymeric interaction at high EPS concentration [29]. 

The forms of EPS that exist outside of cells can be subdivided into bound EPS (sheaths, capsular polymers, condensed gels, loosely bound polymer and attached organic materials) and soluble EPS (soluble macromolecules, colloids, and slimes) [36]. Bound EPS are closely bound to cells while soluble EPS are weakly bound to cells or dissolved in the surrounding solution. Soluble EPS are sometimes referred to as soluble microbial products (SMP) [37]. Soluble EPS have greater binding capacity for organic matter than bound EPS [38]. 

EPS in microbial aggregates have many charged groups (e.g., carboxyl, phosphoric, sulfhydryl, phenolic and hydroxyl groups) and apolar groups (e.g., aromatics and aliphatics in proteins, and hydrophobic regions in carbohydrates) [39]. Thus they have both wetting and cross-linking characteristics, containing both hydrophilic and hydrophobic sites on their structure, which enable them to deposit on both hydrophilic and hydrophobic surfaces. The hydrophobic parts of EPS promote selective adsorption of organics from water [40]. In wastewater, some organic matter can be adsorbed to the EPS matrix, and the hydrophilicity/hydrophobicity of EPS significantly influences the overall hydrophobicity of microbial aggregates in bioreactors [41]. Proteins, carbohydrates and nucleic acids in EPS also have the ability to complex with heavy metals [42]. 

While the microbial cells are important in biofouling as they aggregate on the membrane surface, the decline of membrane permeability is influenced more by the formation and accumulation of EPS on the membrane surface. As the EPS accumulate, the deposited material forms a gel layer by cross-linking with the membrane surface. The gel layer then provides an environment that is rich in nutrients, ideal for further bacterial attachment. The attachment of EPS to form a gel matrix on membrane surfaces depends on a number of factors such as (a) cohesion characteristics of EPS; (b) flexibility and rearrangement characteristics of EPS; (c) adhesion characteristics between EPS and membrane; (d) morphology of membrane surface; (e) diffusion of EPS into the porous surface structure and (f) flow patterns near the membrane surface [43]. The bonding of EPS on the membrane surface strengthens over time due to the flexibility and cross-linking character of EPS. 

The biofouling potential of EPS is significantly greater than some types of natural organic matter and the permeate flux decline during biofouling is strongly related to the EPS content [44]. EPS reduce turbulent flow near the membrane surface, leading to elevated concentration polarization. They also reduce the void fraction between cells in the biofilm, resulting in reduced water permeation through the membrane [45]. The reactivity of EPS with solutes hinders the back-diffusion of solutes (from the membrane surface to the bulk phase across the biofilm), contributing to the increase of trans-membrane osmotic pressure and permeate flux decline in NF and RO systems [46]. During membrane cleaning, EPS act as a diffusion barrier, retarding convective flow and transport of anti-microbial agents to microorganisms inside the biofilm [47]. 

Soluble EPS (or SMP) have been shown to be among the most recalcitrant naturally occurring organic foulants of ultrafiltration (UF), NF and RO membranes [48] as they can accumulate on the membrane surface or penetrate into the membrane pores [49]. Protein and protein-like extracellular matter, polysaccharides and polysaccharide-like materials were found to be the prominent foulants on membranes in low-pressure membrane filtration of biologically treated effluent [50]. Polysaccharide- and protein-like matter occur in both macromolecular and colloidal forms. Polysaccharide-like foulants are neutral in character, they interact with the membrane surface through either hydrogen bonding or in a colloidal form, to form a cake/gel layer on the membrane surface. The amphoteric nature of protein-like matter enables it to interact with the membrane surface through either dipole interactions or by forming a cake/gel layer in colloidal form [3]. The characteristics of SMPs and the degree of microbial adhesion to membrane surfaces vary with species, the growth phase and nutritional conditions such as water chemistry, pH and temperature [51]. 

### 2.4. Biofilm Characterization Techniques

#### 2.4.1. Epifluorescence, Confocal Laser Scanning and Electron Microscopy

The use of microscopy methods in biofilm examination has increased over recent years due to the possibility of coupling them with automated digital on-line image acquisition and image analysis [52]. Common microscopy methods for morphological observation of biofilms include epifluorescence microscopy (EFM), confocal laser scanning microscopy (CLSM) and electron microscopy [53]. EFM coupled with staining methods can provide information on microbial activity, total cell counts and the 2-dimensional distribution of bacteria in the biofilm [53]. CLSM provides information about the 3-dimensional structure of biofilms and has the ability to identify different components of the biofilms either by autofluorescence (for algae) or by using specific fluorescent dyes (for bacterial DNA or EPS glycoconjugates) [54]. Electron microscopy methods such as scanning electron microscopy (SEM) and transmission electron microscopy (TEM) have been employed for elucidating biofilm structure [55]. SEM is capable of imaging complex structures of the biofilm while TEM can visualise the cross-sectional detail of individual microorganisms and their relationship to each other [56]. Due to the high vacuum conditions required for SEM, sample preparation such as drying and coating of the samples with a conductive material is necessary. Environmental scanning electron microscopy (ESEM) can be used for the observation of hydrated samples (*i.e.*, in their natural state) and it does not require such sample preparation. ESEM has been used to observe polysaccharide (alginate) fouling on a microporous membrane by Le-Clech *et al.* [57]. 

#### 2.4.2. Scanning Transmission X-Ray, Atomic Force, Soft X-Ray and Digital Time-Lapse Microscopy

Scanning transmission X-ray microscopy (STXM) can be used for examining hydrated biofilms due to the ability of soft X-rays to penetrate water. Lawrence *et al.* [58] have employed STXM, CLSM and TEM to map the distribution of macromolecular subcomponents (e.g., polysaccharides, proteins, lipids, and nucleic acids) in a biofilm and demonstrated that this combination of multi-microscopy analysis can be used to create a detailed correlative map of biofilm structure and composition. 

Other microscopy methods such as atomic force microscopy can be used for eliciting biofilm surface topography [59] or analyzing the EPS on the surfaces of bacterial biofilms [60]. Soft X-ray microscopy can be used for elucidating the initial steps of bacterial colonization [61]. Digital time-lapse microscopy can be used for in-situ study of growth and detachment of biofilms in flow cells [52], and near-field scanning optical microscopy can be used for examining the bacterial community composition and structures of biofilms [62].

#### 2.4.3. Fourier Transform Infrared, Nuclear Magnetic Resonance and Raman Spectroscopy

Fourier transform infrared (FTIR) spectroscopy has been employed for analyzing microbial aggregates on membrane surfaces [63,64] and can provide information about the chemical nature of the fouling layer [64]. It allows one to distinguish the different kinds of fouling on the same membrane but cannot provide information about biofilm thickness [65]. 

Nuclear magnetic resonance (NMR) microscopy has been utilized to study biofouling of industrial spiral wound RO modules [66]. NMR microscopy can provide a non-invasive quantitative measurement of RO membrane biofouling and its impact on hydrodynamics and mass transport in RO systems. 

Recently, Cui *et al.* [67] showed that surface-enhanced Raman spectroscopy (SERS) could be used as a new and versatile tool for examining the fouling of protein on polyvinylidene fluoride (PDVF) membranes. The fouled area can be visualized by a combination of Raman mapping and silver staining. The fouling potential of different proteins could be identified by comparing their relative SERS intensities on a glass slide before and after the mixture was filtered through the PVDF membrane. 

#### 2.4.4. Other Characterization Techniques

Microbial analysis such as heterotrophic plate counts, total direct cell counts and physical and (bio)chemical analysis such as total wet weight deposits, adenosine triphosphate, EPS and proteins have been used to determine the active biomass on spiral wound membranes [68,69]. Bereschenko *et al.* [70] employed a combination of molecular (such as fluorescence *in situ* hybridization, denaturing gradient gel electrophoresis) and microscopy techniques to study the biofilm formation by *Sphingomonas* spp. on RO membranes. However, these techniques can only be used when doing a destructive membrane autopsy. Characterization of EPS is of great interest in biofouling studies as it can provide direct information for biofouling evaluation. The available techniques for EPS characterization are summarized in Section 2.5.

Biofoulants can be detected and identified with different microscopy and other techniques. Microscopy methods usually require the disassembly of the membrane module [63]. Consequently, the biofilm attached to the membrane surface can be disturbed, and once disassembled, the module is destroyed and no longer operable. There is a general lack of information on the quantitative relationship between the biomass concentrations and the resulting operational problems in NF/RO membranes as discussed by Hijnen *et al.* [69]. In that paper, they demonstrated that there was a quantitative relationship between two major cell constituents, ATP and carbohydrate, and the pressure drop. 

### 2.5. Characterization of EPS

Characterization of EPS usually involves its extraction from the biofilm and quantification of the components. EPS extraction is important for studying the physicochemical properties and their impact on contaminants in the aquatic environment. A good EPS extraction method should not alter its characteristics or cause cell lysis, and should collect all of the EPS components [71]. Techniques for EPS extraction can be divided into three categories: physical and chemical methods, and a combination of the two. Common physical methods include centrifugation, dialysis, filtration and sonication [72], ion exchange [73] and heating [74]. Chemical methods utilize chemical agents such as ethylenediamine tetraacetic acid (EDTA), formaldehyde, sodium hydroxide and ethanol for the extraction of EPS from the microorganisms [75]. Physical methods usually yield less than chemical methods although they have the advantage of minimal contamination from reagents and minimal cell lysis [72]. The combination of chemical and physical methods is more effective as a high yield can be obtained without excessive contamination and cell lysis due to the reagents [71].

There are numerous techniques available for analyzing and quantifying EPS components such as colorimetry [76], FTIR spectroscopy, X-ray photoelectron spectroscopy, high performance size exclusion chromatography [77], high performance liquid chromatography [78], gas chromatography-mass spectrometry [79], deoxyribonucleic acid (DNA) assays [80] and proton nuclear magnetic resonance [81]. 

## 3. Monitoring of Membrane Biofouling

Good monitoring systems are necessary for the development and optimization of efficient anti-biofouling strategies. Monitoring techniques should be able to identify the location, the composition and kinetics of the growth of the biofilm. The information on biofouling should be acquired on-line, in-situ, non-destructively, in real time, representatively, accurately, reproducibly, automatically [82] and quantitatively related to the performance of the membrane process. Techniques include monitoring the characteristics of the feed water which indicate or promote microbial growth, detection of biofilm formation and system performance analysis.

### 3.1. Biological Parameters of the Feed Water

Biological parameters such as ATP content, a constituent of all living cells central to energy transfer [83], total direct cell counts (TDC), which represent the concentration of microorganisms [84] or assimilable organic carbon (AOC), substances which promote microbial growth and, the biofilm formation rate (BFR) [85] may be used for assessing the biofouling potential of the feed water. However, these parameters are not suitable for onsite early warning of membrane biofouling as they cannot be determined directly without sampling [68]. The combination of the microbiological parameters ATP, AOC and BFR, however, can be used to detect problematic biofilm formation before pressure drop increase has occurred and as such can be regarded as off line early warning tools [86]. 

### 3.2. System Performance Analysis

Pressure drop, oxygen uptake, permeate flux and salt passage have been used as proxies for biofilm growth on membrane surfaces and so can be used as an early sign of membrane biofouling [87]. As oxygen consumption during membrane operation may be too small to be detected on-line, measurement of oxygen consumption is usually carried out before and after circulation of feed water through a membrane unit for a fixed period of time (e.g., 2 h) or before and after a fixed period of standstill of the membrane unit (e.g., 2 h). The technique has the advantages of being specific for active biomass, applicable in-situ, non-destructive and more sensitive than pressure drop measurement [88]. 

### 3.3. Silent AlarmTM System

MASAR Technologies Inc. has developed the Silent AlarmTM system for early discovery of RO and NF membrane fouling and real-time monitoring of plant operation of the flux decline trend via a normalizing system operating in accordance with the standard method ASTM D-4516 [89]. The technology is capable of detecting the very early stages of fouling or scaling using a parameter known as Fouling Monitor (FM), which represents the percentage differential between the industry standard ASTM-normalized flow and the correct-normalized flow for each data point. A FM of 5% indicates that fouling is starting to develop while a value of over 20% implies that irreversible fouling is occurring. The technology has been found to be effective in monitoring membrane fouling in two major brackish and seawater RO plants in the Arabian Gulf. However, it cannot differentiate between biofouling and organic/inorganic fouling and thus its assistance in the development of an effective strategy for biofouling control is limited.

### 3.4. Fluorometry

In the fluorometry method [90], a fluorogenic agent such as 4-methylumbelliferyl phosphate or pyranine phosphate is added to the feed water. The fluorogenic agent interacts with microorganisms causing a change in the fluorescent signal which is then detected by an on-line fluorometer. To be successful, it is necessary that the fluorogenic agents do not reduce the efficacy of pretreatment chemicals, are compatible with membrane materials, and are environmentally benign. 

### 3.5. Ultrasonic Time-Domain Reflectometry

Ultrasonic time-domain reflectometry (UTDR) has been used for monitoring of early-stage biofilm growth on polymeric surfaces [91]. UTDR uses sound waves to locate a biofilm on the membrane surface and provides information on the physical characteristics of the biofilm through which the waves travel and are reflected [92]. Most studies on membrane biofouling monitoring by UTDR have been carried out at laboratory scale using model solutions of substances such as bovine serum albumin or other proteins [93]; the application of this technique for monitoring of biofouling at industrial scale requires further investigation. 

### 3.6. Biosensors/Nano-Sensors

Biosensors/nano-sensors for the detection of membrane biofouling in seawater reverse osmosis (SWRO) have been investigated [94]. However, the fouling of the sensors over a long period of use and the need for frequent calibration of the sensors are the major challenges for this technique to be successful in an industrial plant for routine use.

### 3.7. Electrical Potential Measurement

The variation of electrical potential during cake layer formation can be used as a biofouling indicator [95]. A streaming potential arises when an electrolyte flows through a porous membrane under pressure, causing an electrokinetic flux which leads to an electrical potential difference between the feed and permeate side of the membrane. Hence membrane biofouling can be monitored by electrical potential measurement between the feed and permeate side of the membrane. The electrical potential variation depends on the cake layer properties such as hydraulic resistance, porosity and thickness. The foulant layer normally contains colloids and/or microorganisms hence it may be highly compressible [96]. Consequently, the pressure is likely to modify the cake structure leading to errors in the electrical potential measurement. For this reason, electrical potential measurement for the monitoring of membrane biofouling should be conducted at a constant pressure [97].

### 3.8. Membrane Fouling Simulator

Direct detection of biofouling on RO membranes can be very difficult due to the high pressure applied and the spiral wound modules employed. Vrouwenvelder *et al.* [98] developed an early warning monitoring system for biofouling on spiral wound NF and RO membranes in seawater desalination. The system included a membrane fouling simulator (MFS) to monitor the feed channel pressure drop over an individual membrane element which is susceptible to fouling, a sensitive differential pressure drop transmitter, and a flow controller. In addition to biofouling monitoring, the system can also be used for selecting and optimizing pretreatment and cleaning strategies for spiral wound NF/RO membranes. However, the MFS unit only monitors the development of the pressure drop in the feed channel under conditions without permeate production therefore it does not monitor flux reduction caused by biofouling. Further integration of the MFS unit with real-time numerical analysis or precision monitoring devices is required to obtain information about fouling layer structure or chemical composition of the foulants in real time [99].

### 3.9. Development of Microbial Sensing Membranes

Microbial sensing membranes have been developed recently by Gorey *et al.* [100]. A stimulus-responsive polymer film, which has the potential to expand into a hydrophilic state when the temperature decreases or collapse into a hydrophobic state when the temperature increases, was grafted onto the cellulose acetate membrane surface. By continuously triggering the phase transition of the polymer film, membrane fouling was reduced. Biological recognition molecules (e.g., antibodies) targeting selected bacteria can be covalently bonded to the polymer film for in-situ biofouling detection. This is a significant development for the detection of biofouling on membranes, however the efficacy of the biological recognition molecules for long-term use needs further investigation. 

Although the on-line methods for monitoring membrane biofouling have provided useful information regarding the early formation of biofilms, to date they cannot differentiate between the biomass and other components deposited on the membrane, and cannot provide detailed chemical information about the biofilm, therefore their assistance in the development and optimization of efficient anti-fouling strategies is limited. 

## 4. Biofouling Prevention and Control

### 4.1. Biocide Treatment

The conventional anti-fouling strategy has been to dose feed water continuously with biocides or antimicrobial substances. Different bacteria react differently to bactericides, either due to inherent differences such as unique cell envelope composition and non-susceptible proteins, or to the development of resistance, either by adaptation or by genetic exchange. The efficacy of biofouling control by biocides depends on a number of factors such as: type and level of bioactivity in the system, type and concentration of the biocide used, frequency of dosing (continuous *vs.* shock dosing), contact time, pH and concentration of organics and inorganics in the feed water [101]. Biocide treatment must be followed by high velocity detergent cleaning and flushing to remove the organic debris [102]. The effectiveness of biocide treatment can be determined by microbiological tests [103].

Chlorine is the most widely used disinfectant in water and wastewater treatment. In many cases, chlorine (either as a gas or in the hypochlorite form) cannot be used for membrane treatment because (a) most commercially available polymeric membranes are sensitive to chlorine although new chlorine-tolerant membranes are becoming increasingly available [102]; and (b) due to the production of a large amount of AOC which leads to bacterial growth [104]. Chlorine dioxide has been the most promising alternative to chlorine due to its biocidal effectiveness, lower formation of harmful by-products and its relatively mild effect on polymeric membrane structures. Its main drawback is the material cost and handling problem since it is a gas which cannot be generated on site [102]. Chloramines were also introduced as a viable alternative to chlorine [105]. Chloramines are less reactive and thus more stable than free chlorine, especially at high pH. However, chlorination and chloramination have been found to produce undesirable disinfection by-products in the treated water such as trihalomethanes, haloacetic acids and N-nitrosodimethylamine, some of which are suspected carcinogens and some of which can permeate the membranes. Another issue is that it has been found that certain species of microorganisms produce colonies and spores which agglomerate in spherical or large clusters (e.g., *Bacillus subtilis*) and chlorination of such clusters may destroy the microorganisms on the cluster surface but leave the innermost organisms intact [106]. Several studies have shown that potassium ferrate is an attractive alternative disinfectant to chlorine in water and wastewater treatment [107]. The advantage of using ferrate is that it does not produce any mutagenic by-products during treatment processes [108]. 

Ozone has been used as a disinfectant in water and wastewater treatment due to its powerful oxidation effects. Ozone is effective for deactivating bacteria, viruses, protozoa and endospores. However, due to its instability, ozone must be produced on-site. Ozone can form mutagenic and carcinogenic agents such as bromate in the treated water [109]. Furthermore, ozonation in wastewater treatment leads to a net production of AOC which can be easily taken up by bacteria [110] and thus promote microbial growth.

Other oxidizing biocides which have either been used or considered for use in RO plants include iodine, hydrogen peroxide and peracetic acid. Their use for membrane disinfection is limited due to their oxidative effect on polymeric membranes which increases the permeability and so reduces their effectiveness, and reduces the lifetime of the membranes and so increases the operating cost, but they have been very efficient for disinfecting the pretreatment section components in membrane filtration processes such as pipes, manifolds and other hard-to-reach stagnant flow areas [102]. Sodium bisulphite has been used as a biocide in water and wastewater treatment since it binds oxygen and so makes it unavailable. Its efficiency is dependent on the species of microorganisms [105]; it was found that aerobic marine bacteria and certain microorganisms such as sulphate-reducing bacteria have shown resistance to sodium bisulphite [111]. 

Non-oxidizing biocides such as formaldehyde, glutaraldehyde and quaternary ammonium compounds have been used in water treatment but their long term use may lead to acclimation of the microbes to be resistant and this is a drawback of using biocides in water treatment processes [111].

In general, biocide treatment with chemicals mainly tackles the suspended cells but does not reduce the AOC concentration [112]. Continuous biocide application usually generates waste which can cause environmental, ecological and toxicological problems and increase the treatment cost. Therefore, monitoring and bioassay should be conducted to assess the impact of biocides on the receiving environment and a decision support system needs to be developed as noted by Lattemann [113].

UV irradiation has been used for disinfection in water and wastewater treatment. UV irradiation is a physical process which inactivates and destroys both bacteria and viruses [114]. UV irradiation produces hydroxyl radicals which inhibit microbial growth and reduce the AOC concentration [115] as well as degrade macromolecules to smaller fragments. Irradiation at 254 nm breaks down the DNA and thus inhibits bacterial reproduction. The performance of the UV disinfection process is not only determined by the microbial reduction kinetics, but also by the spatial distribution of microorganisms and the UV intensity [116]. Although it reduces the viable organism count, UV disinfection cannot control biofouling within the membrane modules. The main advantages of UV treatment include its simplicity, no need for chemical addition, minimal space requirement, short contact time and fewer harmful by-products [117]. However, its relatively high cost and the difficulty of optimizing and monitoring dosages have limited its applicability to mostly small, fully automated systems [102]. The efficacy of UV irradiation is limited in highly light scattering [118] or UV-absorbing solutions or when the microorganisms are capable of photo-reactivation [119].

### 4.2. Nutrient Limitation

Amon *et al.* [120] reported that both low molecular weight (LMW < 1 kDa) and high molecular weight (HMW >1 kDa) dissolved organic compounds can be utilized by bacteria, however, HMW dissolved organic carbon (DOC) was utilized to a greater extent than LMW DOC. Biodegradable organic matter can be classified into biodegradable dissolved organic carbon (BDOC) and the previously mentioned AOC. BDOC is a macro-parameter which is related to LMW and HMW biodegradable compounds in water. BDOC can be used to define water biostability: water is considered as biologically stable when the BDOC concentration does not exceed 0.15 mg C/L in unchlorinated water [121]. However, the role of BDOC as a measure of biological stability is controversial. Charnock and Kjønnø [122] found no correlation between AOC and BDOC in raw water and drinking water, and noted that these parameters are independent measures of different biodegradable organic fractions. Van der Kooij [85] suggested that BDOC could not be used to predict the regrowth level because no significant correlation was found between BDOC and heterotrophic bacteria counts. However, AOC concentration is one of the most important factors in controlling attached biomass and heterotrophic bacteria activity in water as AOC is the part of DOC that can be easily assimilated by bacteria and converted to cell mass.

The AOC is quantitatively related to the microbial growth and biofouling in spiral wound membranes [28]. For systems that are experiencing bacterial regrowth problems, three different AOC levels have been proposed to limit the growth of heterotrophic plate count bacteria in unchlorinated systems: (a) less than 100 μg/L as suggested by Le Chevallier *et al.* [123]; (b) a level similar to that of groundwater (about 50 μg/L) as proposed by Bradford *et al.* [124] because bacterial regrowth problems are rarely experienced in groundwater; and (c) less than 10 μg/L, as recommended by van der Kooij [125]. The first two levels can be achieved by using conventional treatment processes, but the third level would be very difficult to achieve by conventional treatment [126]. According to Hijnen *et al.* [28], the threshold concentration of AOC in feed water for biofouling of spiral wound membranes is about 1 µg/L. As the measurement of AOC must be carried out in very clean laboratory conditions it cannot be determined and so used for routine monitoring in the field, but has application in diagnosing the microbial growth potential of the feed water in laboratory-based process design and optimization studies. The AOC level in feed water can be reduced by activated carbon adsorption [127], biological filtration [128], slow sand filtration [129] or membrane filtration [130]. Granular activated carbon helps to reduce the AOC content to a limited degree and its efficiency increases when combined with biological processes (biological activated carbon filter) [104]. A very important aspect in controlling biofouling is the quality of chemicals used for pretreatment of feed water which can contain low amounts of easily biodegradable compounds as demonstrated by Van der Kooij *et al.* [86].

Phosphorus or phosphate limitation can inhibit microbial growth in water. Phosphate limitation can be used to control biofouling in spiral wound reverse osmosis membranes in full-scale installations [131]. Phosphate removal from water and wastewater can be achieved through chemical precipitation [132], crystallisation [133], ion exchange [134] and adsorption [135]. Chemical precipitation, which uses coagulants such as alum, lime, iron salts and polyelectrolytes [136], has some disadvantages such as high maintenance cost, sludge handling and disposal problems, and the need for neutralization of the treated water [137]. 

Electrochemical coagulation for phosphate removal has been investigated by Vasudevan *et al.* [138]. Usually, aluminium or iron plates are used as electrodes and metallic ions generated from these electrodes undergo hydrolysis near the anode to produce activated hydroxide intermediates which are able to destabilize the finely dispersed particles in the water/wastewater. The destabilized particles then aggregate to form flocs. Advantages of the electrocoagulation process include its high particulate removal efficiency, compact treatment facility with relatively low cost and the possibility of complete automation [139]. 

Adsorbents such as blast furnace slag, dolomite, red mud and fly ash have also been used for phosphate removal [140]. The main drawbacks of using these adsorbents are low removal efficiency and high costs [138]. In a biological treatment plant, phosphate removal can be achieved through transferring phosphate in the liquid to the sludge phase. However, the removal efficiency does not exceed 30% therefore another technique is required for removing the remaining phosphate [141]. If phosphorus levels are problematic, the use of phosphorus-containing antiscalants should be avoided. 

### 4.3. Other Biofouling Control Methods

#### 4.3.1. Biological Controls

Microorganisms can use quorum sensing (QS) to co-ordinate their communal behavior, e.g., biofilm formation, swarming, motility and production of EPS [142]. The QS-coordinated process is achieved by releasing and detecting small signal molecules known as auto-inducers (AIs). QS systems play an important role in the regulation of microbial attachment and subsequent biofilm formation. Three types of AIs have been identified, namely oligopeptides, N-acylhomoserine lactones (AHL), and autoinducer-2 (AI-2) synthesized by the LuxS gene [143]. Oligopeptides and AHL are involved in cellular communication for Gram-positive and Gram-negative bacteria respectively, whereas AI-2 is universal for inter-species communication for both Gram-positive and Gram-negative bacteria [144]. QS inhibition would provide some means to control biofilm growth without the use of growth-inhibiting agents [145]. 

Kim *et al.* [146] found that 60% of bacterial species on fouled RO membranes collected from a water treatment plant produced QS molecules. These microorganisms were actively involved in biofilm formation on membranes, suggesting that biochemical control of biofilm formation by inhibiting QS signals could be an effective way to reduce membrane biofouling. Ponnusamy *et al.* [147] found that 2(5-H) furanone can be used for suppressing biofilm formation by environmental strains of bacteria, such as *Aeromonas hydrophila*, isolated from a bio-fouled RO membrane system. Kappachery *et al.* [148] demonstrated that a commercially available vanillin (4-hydroxy-3-methoxybenzaldehyde) can be used to prevent the establishment of biofilm on RO membrane surfaces. Yeon *et al.* [149] demonstrated that membrane biofouling due to a mixed culture of Gram-positive and Gram-negative bacteria could be efficiently mitigated by the use of AHL inhibitors such as Acylase I. The applicability of QS to biofouling in membrane filtration in water and wastewater treatment remains a challenge and the effectiveness of the QS inhibitors for biofouling control in membrane systems at industrial scale requires further investigation.

Another biological control technique is the use of bacteriophage which can be used to inhibit or disrupt biofilm development on membrane surfaces. Bacteriophages infect the host bacteria and can undergo rapid replication of virions and lyse the host cells [150]. Goldman *et al.* [151] demonstrated that the addition of specific bacteriophages to a membrane bioreactor could reduce microbial attachment to the membrane surface and increase the membrane permeability. When multiple species of contaminant bacteria are present, a combination of several phage types may be needed to prevent adhesion and biofilm formation on the membrane surface [152]. Since the pore size of UF, NF and RO membranes is generally smaller than the phages, some of the seed phages will be left attached to the membrane surface and so available for continuous infection of the oncoming bacteria with no or negligible interference to the filtration process [153]. However, the specific parasitic characteristics of bacteriophage would eventually pose a challenge to their application in large-scale wastewater treatment [143].

Nitric oxide (NO) has been identified as an important messenger molecule that regulates biofilm dispersal. For example, addition of NO at low, non-toxic concentrations results in the dispersal of a biofilm of *Pseudomonas aeruginosa* [154]. NO was found to have a universal effect on dispersal of sessile bacteria, including both Gram-positive and Gram-negative bacteria [155]. Therefore, NO would have great potential for controlling microbial attachment and membrane biofouling. However, as NO has low solubility in water and is easily oxidized, direct addition of NO into aqueous solution would make it less effective in biofouling control [156]. Many NO donors, including the enzymatic and non-enzymatic NO donors (e.g., sodium nitroprusside, 3-morpholinosydnonimine, sodium nitrite, S-nitroso-N-acetylpenicillamine and diazeniumdiolate) have been proven to be efficient in the dispersal of biofilms. The addition of a NO donor, such as sodium nitroprusside, increased the biofilm removal efficiency for RO membranes, and the dispersal of multi-species biofilms from water and treatment systems was induced by various NO donors at picomolar or nanomolar levels [154]. The NO-based method for biofouling control is still at an early developmental stage and restricted to *in vitro* studies, therefore, more effort is needed to further explore its potential in membrane biofouling at pilot scale [143].

#### 4.3.2. Electrokinetic Methods

Electrical fields have been used to enhance water transport through membranes [157]. Brunner and Okoro [158] demonstrated that an electrical field can reduce membrane fouling during ultrafiltration of protein solutions. This is due to the gel layer being completely removed by electrophoresis, and electro-osmosis acts as an additional driving force for the water flux. In addition to these phenomena, electrolysis, Joule heating and ion migration also occur. One or more of these mechanisms occur and improve membrane performance [159]. Brors [160] found that alternating fields have a greater beneficial effect on the microfiltration of microbial suspensions than DC fields. The alternating field induces a vibration of the particles in the gel layer and in the pores, thus fouling is reduced and the permeate flux is increased. Zumbusch *et al.* [161] applied alternating electrical fields in the ultrafiltration of biological suspensions and found that the alternating electric field could diminish membrane fouling and thus yielded higher specific permeate flux. One of the major hurdles of the commercial application of this technique is the availability of suitable corrosion-resistant and inexpensive electrode material. The success of the electrokinetic technology in biofouling control also depends on the development of novel types of material, which not only behave as membranes but also have the ability to conduct electricity. Currently, there are some ceramic materials available which can conduct electricity but only at high temperatures [159].

#### 4.3.3. Pretreatment of Feed Water with Coagulants

Coagulants have been used in water and waste water treatment for removing colloidal and soluble organic materials for many years. Coagulation changes the particle characteristics such as size, charge, and shape which can be related to the improvement of permeation rates and/or in permeate quality. Coagulation can substantially reduce the concentration of biodegradable organic matter in water hence it can reduce the potential for biofouling [162]. Coagulation, especially when followed by sedimentation, can remove considerable amounts of foulants [163]. At high coagulation doses, coagulation/flocculation can remove polysaccharide-like and protein-like matter, but subsequent clarification using adsorbents may be required. Shon *et al.* [164] found that flocculation followed by adsorption using granular activated carbon could result in a removal of effluent organic matter of more than 90% and significantly improved the permeate flux for ultrafiltration of biologically treated sewage effluent. 

Aluminium-based and iron-based coagulants were found to be effective in enhancing the filterability of mixed liquor and controlling fouling in membrane bioreactors [165]. Tran *et al.* [166] showed that commercial polysilicato-iron (PSI) can reduce biopolymer (protein, carbohydrate) concentrations and membrane biofouling in membrane bioreactors through the coagulation of SMP. Wu *et al.* [167] found that polymeric coagulants such as polymeric ferric sulphate could be used for controlling membrane fouling in submerged membrane bioreactors by reducing the initial trans-membrane pressure and its rate of increase. They proposed three functions of polymeric coagulants in membrane fouling control: (a) restricting the formation of the gel layer on membrane surface; (b) slowing the development of biofilm and (c) assisting the removal of stable foulants from the membrane surface. 

Coagulation of algal cells can be difficult due to their widely variable physical and chemical characteristics [168]. Complex cell morphologies such as spiny appendages prevent close contact of cells [169] and motility enables cells to escape from the flocs [170]. Algogenic (algal) organic matter prevents agglomeration and increases the negative charge at the cell surface and thus increases the coagulant demand [171]. Consequently, coagulant demand cannot be calculated on a stoichiometric basis [172]. 

Frequency of membrane cleaning depends on the success of coagulation. It was found that the coagulated water still has high biofouling potential [173] and the coagulant residuals from the coagulation process can have a negative effect on the performance of the membranes [174]. The success of feed pretreatment by coagulation depends on the nature of the feed water, the membrane type and configuration, the water recovery expected and the frequency of membrane cleaning. Feed pretreatment generally has only a temporary effect on reducing biofouling. Microorganisms can survive pretreatment processes such as coagulation, flocculation and sand filtration and, with time, they will continue to colonise a variety of surfaces within the treatment plant [175]. It was found that pretreatment by microfiltration (MF) or UF prior to RO in wastewater treatment is more effective compared with coagulation due to better resultant water quality of the RO feed water and the use of high coagulant doses can be avoided [176].

#### 4.3.4. Treatment with Silver Nanoparticles

The antimicrobial properties of silver compounds and silver ion (Ag^+^) have been utilized in a wide range of applications including water systems [177]. Silver nanoparticles (Ag-NPs) have been shown to be very effective in controlling the growth and activity of various microorganisms [178] and inactivating planktonic *Escherichia coli* in aqueous suspensions, and so Ag-NPs can be used for retarding biofilm formation on surfaces in water. However, they are not effective for eradicating existing or mature biofilms or use as a disinfectant [179]. This pretreatment method has not been widely employed in membrane processes and it may not be a cost-effective choice for membrane filtration of water and wastewater treatment at an industrial scale due to the large quantity of Ag-NPs which would be required for the treatment of the feed water. 

#### 4.3.5. Membrane Surface Modification

The traditional method for biofouling control has been the selection of membrane materials which have low bacterial affinity or can be easily cleaned. Another approach is to give the membrane bacteriostatic properties through membrane surface modification which can effectively inhibit the growth of microorganisms [180]. 

*Surface modifications:* Surface modifications can be achieved through polymer blending, grafting, coating or using inorganic or antimicrobial additives during membrane manufacture.

- *Polymer blending approach*: Polymer blending changes the surface characteristics with only minor alteration of the bulk morphology and properties of the membrane [181].
- *Grafting approach*: The grafting technique employs hydrophilic polymers or plasma treatment to produce anti-fouling membrane surfaces [182]. Although this technique can be applied for any polymeric material, most of the recent work on membrane surface graft polymerization was carried out on thin-film composite polyamide membrane or porous polypropylene membrane [183].
- *Surface coating*: Surface coating with additives is a simple method which can be easily adapted to existing membrane manufacturing processes. After coating, the surface properties of the membrane such as hydrophobicity, roughness and surface charge are modified and the resistance to biofouling improved.
- *Inorganic additives*: Inorganic additives used for improving the antifouling properties of membranes include nano-sized titanium dioxide [184], silica [185], nano-sized alumina [186], zirconium dioxide [187] and lithium perchlorate [188]. Most work on titanium dioxide has been focused on the use of UV irradiation to improve the antifouling property of the membranes through photocatalytic degradation of the foulants prior to reaching the membrane surface [189].
- *Antimicrobial additives*: Additives which have been used for giving membrane surfaces antimicrobial properties include synthesized antimicrobial polymers which contain quaternary ammonium or phosphonium salts [190], polyethylene oxide [191], heavy metals such as copper [192] or silver [193], chitosan [194], and silver nanoparticles (Ag-NPs) [195]. Hybrid NF and UF membranes with immobilized Ag-NPs have shown good antibiofouling characteristics [196]. Recently, Chae *et al.* [197] showed that membranes coated with fullerene nanoparticles (C60) led to decreased microbial attachment and inhibited respiratory activity, therefore the biofouling resistance of membranes coated with C60 would be enhanced. However, as their study focused on the growth of *E.coli* K12 (cultured in Luria Bertani broth) on a MF membrane coated with fullerene nanoparticles, the effectiveness of the system for membrane fouling control for use with water and wastewater which contain many different microbial species requires further investigation. The EPS released from the microorganisms in the biofilm can be a barrier which can hinder the contact between the microorganisms in the biofilm and antimicrobial additives. Thus the anti-adhesion approach which prevents the initial attachment of the microorganisms to a membrane surface should be a more effective method than the antimicrobial approach which aims at killing microorganisms already attached to the membrane.

*Low-protein fouling membranes:* As most proteins and cells are negatively charged in aqueous solution [14], introduction of negative charges on the membrane surface should increase the electrostatic repulsion between the membrane and cells/proteins and thus reduce biofouling on membranes. There has been considerable research on developing membrane surfaces that can effectively inhibit protein adsorption. To achieve high resistance to protein and microbial fouling by both positively and negatively charged species, the membrane surface should be highly hydrophilic and have overall neutral charge. Zwitterionic polymers such as polyphosphobetaine, polysulfobetaine, and carboxybetaine have been used for modifying membrane surfaces to improve their resistance to protein adsorption [198]. Zwitterionic polymers are biomimetic materials in which cationic and anionic groups are located on the same monomer and thus maintain overall charge neutrality. They are recognized as a unique type of material that has excellent antifouling properties because of their strong electrostatic interaction with the membrane surface to form a hydration layer which resists protein adsorption [199]. 

Zwitterionic materials based on 2-methacryloyloxyethyl phosphorylcholine have been used for improving the biocompatibility of membranes through coating and blending methods [200]. However, phosphorylcholine-containing polymers lack long-term stability due to the hydrolysis of the phosphoester groups [201], and phosphorylcholine-based monomers are moisture sensitive and are not easy to synthesize and handle [202]. Polyethersulfone and polyacrylonitrile UF membranes with low fouling properties were prepared by Shi *et al.* [203] through a phase inversion method using sulfobetaine-containing polymers. PVDF ultrafiltration membranes with good anti-fouling properties through grafting sulfobetaine methacrylate polymer onto the membrane surface have been developed by Chiang *et al.* [204]. A highly hydrophilic and low-protein fouling polypropylene membrane was obtained by surface modification with sulfobetaine methacrylate [205]. 

Polypropylene membranes grafted with copolymer brushes through UV-induction of two opposite charged monomers such as [2-(methacryloyloxy)ethyl]trimethylammonium chloride and 3-sulfopropyl methacrylate potassium salt were found to have the ability to resist both protein adsorption and biofilm formation [206]. Poly(ethylene glycol) (PEG) has been shown to improve membrane resistance to nonspecific protein adsorption. However, PEG is susceptible to oxidative degradation and chain cleavage in aqueous systems, especially in the presence of transition metal ions [207]. Grafted PEG brushes on the membrane surface were found to lose their resistance to protein adsorption at temperatures above 35 °C [208]. Permanent surface modification by grafting hydrophilic groups often results in change of the membrane structure and integrity [209].

Brink and Romijn [210] coated a range of surface-active compounds onto polysulfone UF membranes from bulk solution in a pretreatment process and they found a significant reduction of protein adsorption on the surfactant-modified membranes. It has been reported that the use of small charged surfactants for membrane modification had limited success when used in pretreatment, possibly due to the displacement by protein or its solubilization into the bulk solution during filtration [211]. Chen *et al.* [209] employed a mixed surfactant consisting of bulky nonionic and small anionic surfactants to improve the resistance of polysulfone membrane to protein. The bulky ionic surfactant prevents protein from aggregating near the pore entrance (steric hindrance) while the small anionic surfactant reduces the fouling potential of those proteins which could penetrate the first non-ionic surfactant layer through electrostatic repulsion. However, the long-term stability of the coated membranes requires further investigation.

Extensive studies have focused on generating membranes which can decrease microbial attachment through surface modification such as polymer blending, graft polymerization and coating with inorganic or antimicrobial additives. Appropriate surface modification can slow down the biofilm formation and thus reduce frequent chemical cleaning or biocide treatment. The main issues associated with the polymer blending approach are the miscibility of polymer pairs and the stability of the modified surfaces. Surface grafting inevitably leads to permanent change of membrane chemistry, permeability and pore size [212] which would affect the overall performance of the membrane and the quality of the water product. Many coatings do not have long term mechanical and chemical stability and there is potential for the delaminating of the coating material during chemical cleaning. 

Effective anti-adhesion surface properties plus antimicrobial surface functions appear to be the best solution to control and minimize membrane biofouling [198]. The synthetic antimicrobial polymers and other membrane additives may face strict environmental regulatory hurdles, and bacteria may develop resistance to non-oxidizing antimicrobial compounds. 

#### 4.3.6. Module Design and Optimal Hydrodynamic Conditions

Spiral wound modules (SWMs) are the most commonly used modules in water and wastewater treatment. The performance of an SWM is affected by many factors such as module and spacer design modifications, fouling propensity and ability to be cleaned, and operating conditions [213]. In general, membrane fouling of SWMs can be controlled either by increasing shear rate (velocity) or turbulence near the membrane surface. Shear rate can be increased by pumping the feed at a higher flow rate or by using thin flow channels above the membrane surface, while turbulence can be promoted by appropriate design of feed spacers or the use of static mixers [214]. However, increasing turbulence would increase nutrient supply to the biofilm. For an established biofilm, high shear rate would result in a more compact and less filamentous biofilm structure, and a biofilm which was formed at high shear rate is difficult to remove [215].

In an MBR, optimization of the hydrodynamic conditions is essential for preventing biofouling or at least reducing the biofouling rate. This can be done by controlling the aeration intensity and time in submerged MBRs, or controlling the flow velocity of mixed liquor in cross flow MBRs. Optimization of the hydrodynamic conditions in an MBR plant can also achieved by appropriate design of membrane modules and pilot testing to determine the optimal hydraulic conditions [216].

#### 4.3.7. Membrane Cleaning

Membrane cleaning plays a vital role in biofouling control. Membrane cleaning is usually carried out when there is a significant drop in permeate flux or salt rejection, or when the trans-membrane pressure needs to be increased significantly to maintain the desired permeate flux. Cleaning should weaken the attachment of the fouling layer (by interfering with the interactions between microorganisms and the membrane surface) and remove the foulants from the membrane surface (usually performed by shear forces) [217]. Therefore development of an optimum membrane cleaning regime requires understanding of the interactions between the foulants and the membrane surface, the effect of the cleaning procedure on foulant removal, and the performance of the membrane after cleaning [218]. Membrane cleaning can be classified into physical and chemical cleaning with the former including hydraulic, pneumatic and mechanical processes, and electrical field applications, and the latter involving the use of chemicals such as acids, bases, oxidants and surfactants. In practice, physical cleaning followed by chemical cleaning is widely employed for maximizing the effect.

##### 4.3.7.1. Physical Cleaning

Hydraulic cleaning such as flushing and backwashing/back pulsing is the most common technique for mitigating fouling [219]. Regular intermittent backwashing will lift the foulants off the membrane surface and minimize the extent of concentration polarization [220]. Hydraulic cleaning has become the standard cleaning procedure in MBR and other cross flow filtration systems [221]. Rapid backwashing effectively removes the non-adhesive foulants from membrane surfaces and thus reduces reversible fouling. 

Pneumatic cleaning includes air sparging, air lifting, air scouring and air bubbling [221]. Pneumatic cleaning has the benefits of low maintenance cost, is easy to integrate into the membrane system and no chemicals are required. However, the effectiveness of air sparging in membrane cleaning is limited and associated with high pumping costs. The combination of air sparging and hydraulic backwash is commonly applied in MBRs [222] and for controlling biofouling in spiral wound membranes [223]. Mechanical cleaning such as sponge ball wiping has been employed to scrub the foulants from the membrane surface, however this technique is only applicable for cleaning tubular membranes [224]. 

Ultrasound can be used for membrane cleaning as the ultrasound waves create cavitation and induce acoustic streaming which provide vigorous mixing and break-up of the cake layer on the membrane surface [221], and disruption of microbial cells [225]. The cavitational mechanism plays an important role in detaching foulants from the membrane surface while acoustic streaming plays a vital role in moving foulants away from membrane surface after detachment [226]. The efficiency of ultrasound for membrane cleaning and microorganism deactivation is affected by many factors such as ultrasound frequency, power intensity, feed properties, membrane materials, crossflow velocity, temperature and pressure [227]. The advantages of ultrasound cleaning include (a) the membrane can be cleaned whilst in use, (b) there are no harmful by-products and (c) the hydrogen peroxide and hydroxyl radicals produced by ultrasound during cleaning can also act as disinfectants [228]. However, it has been reported that the membrane becomes damaged and this should be taken into account [229]. Ultrasonic cleaning systems are difficult to scale up to pilot or industrial scale [230].

The application of electrical fields to mitigate membrane fouling has been regarded as a physical cleaning method although it is traditionally employed to enhance water transport through the membrane [157]. Tarazaga *et al.* [231] found that electric fields can remove the biomass deposited on the membrane surface and thus restore the initial permeate flux. They developed an electrical model to describe the current intensity during membrane cleaning, which may be useful for automatic control of membrane cleaning.

Self-collapsing air micro-bubbles (with diameters of less than 50 µm) have been shown to be a potential chemical-free cleaning technology for biofilm detachment from membrane surfaces due to their unique capacity to shrink and subsequently collapse in solution [232]. However, this technology has not yet been employed for membrane cleaning at industrial scale. 

##### 4.3.7.2. Chemical Cleaning

Chemicals which are generally employed for membrane cleaning include alkalis, acids, metal chelating agents, surfactants and enzymes. In addition to these five main categories, disinfectants and oxidants or sequestration agents such as hydrogen peroxide and hypochlorite, sodium bisulphite and EDTA are often used for cleaning membranes. 

Common chemical cleaning agents are caustic (NaOH, KOH, NH_4_OH), acidic (HCl, HNO_3_, H_2_SO_4_, H_3_PO_3_, citric, oxalic), sequestering/complexing (EDTA), detergent/surfactant (alkyl sulphate, sodium dodecyl sulphate, cetyl trimethyl ammonium bromide), enzymatic (α-CT, CP-T, peroxidase), oxidants/disinfectants (NaOCl, H_2_O_2_, KMnO_4_) and cleaning blends (e.g., 4 Aqua clean^®^, TRiclean^®^, Ultrasil^®^/Aquaclean^®^) [233]. The function of caustic in membrane cleaning involves hydrolysis and solubilization of the foulants such as proteins and saccharides [234]. The pH of the cleaning solution can be as high as 13 and at this pH, phenolic and carboxyl groups of the foulants are converted into phenolate and carboxylate and thus their solubility greatly increases [235]. Caustic also increases the negative charge of humic substances in the foulant layer and so would weaken the bonds between them and the membrane. Furthermore, the repulsion between negatively charged functional groups creates a loose fouling layer that allows easier access for chemicals to penetrate the inner portion of the fouling layer and thus enhances the cleaning efficiency. Phosphate, citric acid, salts and other ionic compounds have been used for membrane cleaning because they can interfere with the weak electrostatic interactions between microorganisms and the membrane surface [45].

Surfactants can solubilize the foulants by forming micelles around them [236], making them easy to remove from the membrane surface [237]. Surfactants were found to affect the hydrophobic interactions between bacteria and membranes and thus interrupt biofilm formation on the membrane surface [238].

Although EPS are irreversible foulants [239], they can be removed from the membrane surface by traditional physical and chemical methods. It was found that specific enzymes can break down the EPS and thus prevent biofilm formation [240]. These highly selective biocatalysts can be used for removing established biofilms without producing toxic by-products. Two main types of enzymes, proteases (for hydrolysis of proteins) and polysaccharases (for hydrolysis of polysaccharides), can be used for degrading EPS and thus for biofilm detachment from surfaces [241]. For example, proteases such as proteinase K and trypsin have been employed to remove established biofilms [242]. Enzymatic cleaning has some inherent drawbacks which, to some extent, may limit its large-scale application such as: (a) the activity of enzymes is pH-dependent and they are sensitive to temperature and salt concentration; (b) high production costs; and (c) soluble enzymes are difficult to recover from an aqueous medium [243]. Furthermore, EPS normally consist of a mixture of macromolecules therefore their removal from a fouled membrane would require several enzymes [244]. 

Recently 2,4-dinitrophenol (DNP), a metabolic uncoupler, was found to be very effective in enhancing biofilm detachment from membranes. DNP suppresses ATP synthesis and lowers subsequent autoinducer-2 (AI-2) production, and thus attachment of suspended microorganisms to membrane surfaces is substantially reduced [245].

When living within the complex EPS structures, microbial communities are less sensitive to chemical cleaning [32] and mature biofilms have been found to be persistent and difficult to completely eradicate [246]. Consequently, chemical cleaning is generally not effective for completely removing and/or destroying the complex multicellular structures [247]. Rapid regrowth of the surface-attached microbial layer results in a repetition of the biofouling-related system failure. Periodic and more frequent chemical cleaning is, therefore, unavoidable, leading to increased usage of cleaning chemicals and increased production of wastewater [70]. Frequent chemical cleaning also shortens membrane life [111]. 

##### 4.3.7.3. Cleaning Efficiency

The efficiency of cleaning is usually determined by measuring the water flux after cleaning at a defined pressure, temperature and circulation velocity. Madaeni and Mansourpanah [248] assessed the chemical cleaning efficiency of fouled RO membranes by resistance removal and flux recovery. Chen *et al.* [249] evaluated the cleaning efficiency based on three parameters: (a) clean water flux recovery; (b) wash water usage, which is defined as the ratio between the volume of wash water used and total volume of water produced and (c) the improvement in total dissolved solids rejection measured before and after cleaning. Chemical cleaning efficiency depends on the type of the cleaning agent and its concentration, and the operating conditions such as crossflow velocity, pressure, turbulence near the membrane surface, temperature, pH and cleaning time [250]. 

Cleaning strategies must be able to remove biomass from spiral-wound membrane modules. If the feed channel is completely blocked by biomass, this can limit the transport of the cleaning chemicals to the blocked spacer and restrict the removal of biomass from the membrane module. Therefore, early cleaning of partially fouled membranes and isolating the lead membrane modules from the installation is vital [216]. Combination of chemical cleaning and backwash is more effective than chemical cleaning alone. However, it must be remembered that membrane cleaning is only a part of the biofouling control strategy.

## 5. Concluding Remarks

Biofouling represents a complex mechanism where the quality of the feed water, the physico-chemical properties of the membrane and the operating conditions all play a role. Biofouling begins with the attachment of microorganisms to the membrane surface leading to the formation of a biofilm layer. Extracellular polymeric substances excreted from microorganisms play an important role in bio-flocculation and enhancing microbial attachment to membrane surfaces by mechanically cross-linking and stabilizing the biofilm. The complexity of biofilms means that obtaining a good understanding of them is an important step for the development of better biofouling control strategies. The utilization of various microscopy (e.g., SEM, TEM, CLSM, ESEM) and spectroscopy (e.g., FTIR, NMR and surface enhanced Raman spectroscopy) methods for biofilm examination has been increasing. These methods are means to elucidate the factors related to the occurrence of biofouling in order to control the process in practice.

There has been significant expansion of knowledge of biofouling and the development of monitoring and control of biofouling over the last twenty years. We now know for instance that the process of biofouling occurs at very low nutrient concentrations and will always be part of the membrane filtration process as in conventional filter beds. A clear diagnosis of the magnitude of biofouling by feed water analysis and membrane autopsies with appropriate microbial methods in combination with appropriate process design and operations (pretreatment, chemical dosing and membrane design) and an effective cleaning plan should enable the development of an effective biofouling control strategy. There are parameters available for comprehensive monitoring and diagnosis of the biofouling problems but there is still a need for applied scientific research at plant sites to demonstrate the effectiveness of the developed control strategy.

Models used to diagnose and predict biofouling should be developed which can be used for process design and optimization. Biological methods for the control of membrane biofouling such as inhibition of quorum sensing, enzymatic disruption of EPS, cell wall hydrolysis, metabolic energy uncoupling and bacteriophage have potential, however their applications in water and wastewater treatment at large scale require further investigation.

## References

[1] Amjad Z. (1992). Reverse Osmosis, Membrane Technology, Water Chemistry and Industrial Application.

[2] Flemming H.-C., Griebe T., Schaule G., Schmitt J., Tamachkiarowa A. (1997). Biofouling—The Achille’s heel of membrane processes. Desalination.

[3] Amy G. (2008). Fundamental understanding of organic matter fouling of membranes. Desalination.

[4] Komlenic R. (2010). Rethinking the causes of membrane biofouling. Filtr. Sep..

[5] Vrouwenvelder J.S., van der Kooij D. (2002). Diagnosis of fouling problems of NF and RO membrane installations by a quick scan. Desalination.

[6] Kramer J.F., Tracey D.A. The solution to reverse osmosis biofouling. Proceedings of IDA World Congress on Desalination and Water Use.

[7] Abd El Aleem F.A., Al-Sugair K.A., Alamad M.I. (1998). Biofouling problems in membrane processes for water desalination and reuse in Saudi Arabia. Int. Biodeterior. Biodegrad..

[8] Ridgway H.F., Parekh B.S. (1988). Microbial adhesion and biofouling of reverse osmosis membranes. Reverse Osmosis Technology: Application for High Pure Water Production.

[9] Ridgway H.F., Safarik J., Flemming H.-C., Geesey G.G. (1991). Biofouling of reverse osmosis membranes. Biofouling and Biocorrosion in Industrial Water System.

[10] Flemming H.-C., Amjad Z. (1992). Membrane Technology.

[11] Murphy A.P., Moody C.D., Riley R.L., Lin S.W., Murugaverl B., Rusin P. (2001). Microbiological damage of cellulose acetate RO membranes. J. Membr. Sci..

[12] Marshall K.C., Blainey B.L., Flemming H.-C., Geesey G.G. (1991). Role of bacterial adhesion in biofilm formation and biocorrosion. Biofouling and Biocorrosion in Industrial Water System.

[13] Milstead C.E., Riley R.L. (1993). Development of an Improved Cleaning Solution for ROWPU Units; SST Report No.2809–1.

[14] Wilbert M.C. (1997). Enhancement of Membrane Fouling Resistance through Surface Modification. A Study Using the Principle of Membrane Fouling and Cleaning To Develop Ways to Enhance Membrane Fouling Resistance; Water Treatment Technology Program Report No. 22.

[15] Flemming H.-C., Schaule G. (1988). Biofouling on membranes—A microbiological approach. Desalination.

[16] Bendinger B., Rijnaarts H.H.M., Altendorf L., Zehnder A.J.B. (1993). Physicochemical cell surface and adhesive properties of coryneform bacteria related to the presence and chain length of mycolic acids. Appl. Environ. Microbiol..

[17] Costerton J.W., Lewandowski Z., DeBeer D., Caldwell D., Korber D., James G. (1994). Biofilms, the customized microniche. J. Bacteriol..

[18] Marshall K.C., Savage D.C., Fletcher M. (1985). Mechanisms of bacterial adhesion at solid-water interfaces. Bacterial Adhesion.

[19] Park N., Kwon B., Kim I.S., Cho J. (2005). Biofouling potential of various NF membranes with respect to bacteria and their soluble microbial products (SMP): Characterization, flux decline, and transport parameters. J. Membr. Sci..

[20] Daniels S.L., Bitton G., Marshall K.C. (1980). Mechanisms involved in sorption of microorganisms to solid surface. Adsorption of microorganisms to surface.

[21] Mc Eldowney S., Fletcher M. (1986). Effect of growth conditions and surface characteristics of aquatic bacteria on their attachment to solid surfaces. J. Gen. Microbiol..

[22] Ridgway H.F., Rigby M.G., Argo D.G. (1985). Bacterial adhesion and fouling of reverse osmosis membranes. J. Am. Water Work. Assoc..

[23] Donlan R.M., Pipes W.O. (1988). Selected drinking water characteristics and attached microbial population densities. J. Am. Water Work. Assoc..

[24] Sadr Ghayeni S.B., Beatson P.J., Schneider R.P., Fane A.G. (1998). Adhesion of wastewater bacteria to reverse osmosis membranes. J. Membr. Sci..

[25] Kang S.-T., Subramani A., Hoek E.M.V., Deshusses M.A., Matsumoto M.R. (2004). Direct observation of biofouling in cross-flow microfiltration: mechanisms of deposition and release. J. Membr. Sci..

[26] Al-Ahmad M., Abd El-Aleem F.A., Mutiri A., Ubaisy A. (2000). Biofouling in RO membrane systems. Part 1: Fundamentals and control. Desalination.

[27] Flemming H.C., Wingender J. (2010). The biofilm matrix. Nat. Rev. Microbiol..

[28] Hijnen W.A.M., Biraud D., Cornelissen E.R., van der Kooij D. (2009). Threshold concentration of easily organic carbon in feedwater for biofouling of spiral-wound membranes. Environ. Sci. Technol..

[29] Tsuneda S., Aikawa H., Hayashi H., Yuasa A., Hirata A. (2003). Extracellular polymeric substances responsible for bacterial adhesion onto solid surface. FEMS Microbiol. Lett..

[30] Sheng G.-P., Yu H.-Q., Li X.-Y. (2010). Extracellular polymeric substances (EPS) of microbial aggregates in biological wastewater treatment systems: A review. Biotechnol. Adv..

[31] Zhang X.Q., Bishop P.L. (2001). Spatial distribution of extracellular polymeric substances in biofilms. J. Environ. Eng..

[32] Branda S.S., Vik A., Frieldman L., Kolter R. (2005). Biofilms: The matrix revisited. Trends Microbiol..

[33] Mayer C., Moritz R., Kirschner C., Borchard W., Maibaum R., Wingender J., Flemming H.-C. (1999). The role of intermolecular interactions: Studies on model systems for bacterial biofilms. Int. J. Biol. Macromol..

[34] Gómez-Suárez C., Pasma J., Van der Borden A.J., Wingender J., Flemming H.-C., Busscher H.J., van der Maei H.C. (2002). Influence of extracellular polymeric substances on deposition and redeposition of Pseudomonas aeruginosa to surfaces. Microbiology.

[35] Chen V.J., Ma P.X. (2004). Nano-fibrous poly(L-Lactic acid) scaffolds with interconnected spherical macropores. Biomaterials.

[36] Neilsen P.H., Jahn A., Wingender J., Neu T.R., Flemming H.-C. (1999). Microbial Extracellular Polymeric Substances: Characterization, Structure and Function.

[37] Rosenberger S., Kraume M. (2002). Filterability of activated sludge in membrane reactors. Desalination.

[38] Pan X.L., Liu J., Zhang D.Y., Chen X., Song W.J., Wu F.C. (2010). Binding of dicamba to soluble and bound extracellular polymeric substances (EPS) from aerobic activated sludge: A fluorescence quenching study. J. Colloid Interface Sci..

[39] Flemming H.-C., Leis A., Flemming H.-C., Bitton G. (2002). Sorption properties of biofilms. Encyclopedia of Environmental Micrology.

[40] Spath R., Flemming H.-C., Wuertz S. (1998). Sorption properties of biofilms. Water Sci. Technol..

[41] Liu Y., Fang H.H.P. (2003). Influence of extracellular polymeric substance (EPS) on flocculation, settling and dewatering of activated sludge. Crit. Rev. Environ. Sci. Technol..

[42] Priester J.H., Olson S.G., Webb S.M., Neu M.P., Hersman L.E., Holden P.A. (2006). Enhanced oxoplolymer production and chromium stabilization in Pseudomonas putida unsaturated biofilms. Appl. Environ. Microbiol..

[43] Tansel B., Sager J., Garland J., Xu S., Levine L., Bisbee P. (2006). Deposition of extracellular polymeric substances and micro-topographical changes on membrane surfaces during intermittent filtration conditions. J. Membr. Sci..

[44] Fonseca A.C., Summers R.S., Greenberg A.R., Hernandez M.T. (2007). Extracellular polysaccharides, soluble microbial products and natural organic matter impact on nanofiltration membranes flux decline. Environ. Sci. Technol..

[45] Flemming H.-C. (1997). Reverse osmosis membrane biofouling. Exp. Therm. Fluid Sci..

[46] Kim A.S., Chen H., Yuan R. (2006). EPS biofouling in membrane filtration: An analytic modelling study. J. Colloid Interface Sci..

[47] Allison D.G., Maira-Litrán T., Gilbert P., Evan L.V. (2000). Antimicrobial resistance of biofilms. Biofilm: Recent Advances in Their Study and Control.

[48] Her N., Amy G. Identification and characterization of foulants and scalants on NF membranes. Proceedings of the AWWA Membrane Technology Conference.

[49] Meng F., Chae S.-R., Drews A., Kraumer M., Shin H.-S. (2009). Recent advances in membrane bioreactors (MBRs): Membrane fouling and membrane materials. Water Res..

[50] Nguyen T., Fan L., Roddick F.A., Harris J.L. (2009). A comparative study of microfiltration and ultrafiltration of activated sludge-lagoon effluent. Desalination.

[51] Bos R., van der Mei H.C., Busscher H.J. (1999). Physico-chemistry of initial microbial adhesive interactions—its mechanisms and method for study. FEMS Microbiol. Rev..

[52] Stoodley P., Wilson S., Hall-Stoodley L., Boyle J.D., Lappin-Scott M., Costerton J.W. (2001). Growth and detachment of cell clusters from mature mixed species biofilms. Appl. Environ. Microbiol..

[53] Wolf G., Crespo J.G., Reis M.A.M. (2002). Optical and spectroscopic methods for biofilm examination and activity analysis in water and wastewater treatment. Rev. Environ. Sci. Biotechnol..

[54] Neu T.R., Wöelfl S., Lawrence J.R. (2004). Three-dimensional differentiation of phototrophic biofilm constituents by multi-channel confocal and 2-photon laser scanning microscopy. J. Microbiol. Methods.

[55] Blenkinsopp A.S., Costerton J.W. (1991). Understanding bacterial biofilms. Trends Biotechnol..

[56] Surman S.B., Walker J.T., Goddart D.T., Morton L.H.G., Keevil C.W., Weaver W., Skinner A., Hanson K., Cadwell D., Kurtz J. (1996). Comparison of microscope techniques for the examination of biofilms. J. Microbiol. Methods.

[57] Le-Clech P., Marselina Y., Ye Y., Stuetz R.M., Chen V. (2007). Visualisation of polysaccharide fouling on microporous membrane using different characterization techniques. J. Membr. Sci..

[58] Lawrence J.R., Swerhome D.W., Leppard G.G., Araki T., Zhang X., West M.M., Hitchcock A.P. (2003). Scanning transmission X-ray, Laser scanning, and transmission electron microscopy mapping of the exopolymeric matrix of microbial biofilms. Appl. Environ. Microbiol..

[59] Palmer R.J., Sternberg C. (1999). Modern microscopy in biofilm research: confocal microscopy and other approaches. Curr. Opin. Biotechnol..

[60] Hansma H.G., Pietrasanta L.I., Auerbach I.D., Sorenson C., Golan R., Holden P. (2000). Probing biopolymers with the atomic force microscope: A review. J. Biomater. Sci. Polym. Ed..

[61] Gilbert E.S., Khlebnikov A., Meyer-Ilse W., Keasling J.D. (1999). Use of soft X-ray microscopy for analysis of early-stage biofilm formation. Water Sci. Technol..

[62] Ivnitsky H., Katz I., Minz D., Volvovic G., Shimoni E., Kesselman E., Semiat R., Dosoretz C.G. (2007). Bacteria community composition and structure of biofilms developing on nanofiltration membranes applied to wastewater treatment. Water Res..

[63] Ridgway H.F., Kelly A., Justice C., Olson B.H. (1983). Microbial fouling of reverse-osmosis membranes used in advanced wastewater treatment technology: Chemical, bacteriological, and ultrastructural analyses. Appl. Environ. Microbiol..

[64] Suci P.A., Geesey G.G., Tyler B.J. (2001). Integration of Raman microscopy, differential interference contrast microscopy, and attenuated total reflection fourier transform infrared spectroscopy to investigate chlorhexidine spatial and temporal distribution in Canada albicans biofilms. J. Microbiol. Methods.

[65] Schmid T., Helmbrecht C., Panne U., Haisch C., Neissner R. (2003). Process analysis of biofilms by photoacoustic spectroscopy. Anal. Bioanal. Chem..

[66] Graft von der Schulenburg D.A., Vrouwenvelder J.S., Creber S.A., van Loosdrecht M.C.M., Johns M.L. (2008). Nuclear magnetic resonance microscopy studies of membrane biofouling. J. Membr. Sci..

[67] Cui L., Yao M., Ren B., Zhang K.-S. (2011). Sensitive and versatile detection of the fouling process and fouling propensity of proteins on polyvinylidene fluoride membranes via surface-enhanced Raman spectroscopy. Anal. Chem..

[68] Vrouenvelder J.S., Manolarakis S.A., Van der Hoek J.P., van Paassen J.A.M., van der Meer W.G.J., van Agtmaal J.M.C., Prummel H.D.M., Kruithoft J.C., van Loosdrecht M.C.M. (2008). Quantitative biofouling diagnosis in full scale nano filtration and reverse osmosis installations. Water Res..

[69] Hijnen W.A.M., Cornelissen E.R., van der Kooij D. (2011). Threshold concentrations of biomass and iron for pressure drop increase in spiral-wound membrane elements. Water Res..

[70] Bereschenko L.A., Stams A.J.M., Euverink G.J.W., van Loosdrecht M.C.M. (2010). Biofilm formation on reverse osmosis membranes is initiated and dominated by Sphinogomonas spp. Appl. Environ. Microbiol..

[71] Nielsen P.H., Jahn A., Wingender J., Flemming H.-C., Neu T.R. (1999). Extraction of EPS. Microbial Extracellular Polymeric Substances: Characterization, Structure, and Function.

[72] Comte S., Guibaud G., Baudu M. (2006). Relations between extraction protocol for activated sludges extracellular polymeric substances (EPS) and EPS complexion properties. Part I. Comparison of the efficiency of eight EPS extractions methods. Enzym. Microb. Technol..

[73] Frolund B., Palmgren R., Keiding K., Neilsen P.H. (1996). Extraction of extracellular polymers from activated sludge using a cation exchange resin. Water Res..

[74] Morgan J.W., Forster C.F., Evison L. (1990). A comparative study of the nature of biopolymers extracted from anaerobic and activated sludges. Water Res..

[75] Liu H., Fang H.H.P. (2002). Extraction of extracellular polymeric substances (EPS) of sludges. J. Biotechnol..

[76] Dubois M., Gilles K.A., Hamilton J.K., Rebers P.A., Smith F. (1956). Colorimetric method for determination of sugars and related substances. Anal. Chem..

[77] Omoike A., Chorover J. (2004). Spectroscopic study of extracellular polymeric substances from Bacillus subtilis: Aqueous chemistry and adsorption effects. Biomacromolecules.

[78] Garrotte A., Bonet R., Merino S., Simonpujol M.D., Congregado F. (1992). Occurrence of a capsule in Aeromonas-salmonicida. FEMS Microbiol. Lett..

[79] Fox A. (1999). Carbohydrate profiling of bacteria by gas chromatography-mass spectrometry and their trace detection in complex matrices by gas-chromatography-tandem mass spectrometry. J. Chromatogr. A.

[80] Wingenger J.S.M., Rode A., Leis A., Flemming H.C. (2001). Isolation and biochemical characterization of extracellular polymeric substances from Pseudomonas aeruginosa. Methods Enzym..

[81] Harding L.P., Marshall V.M., Elvin M., Gu Y.C., Laws A.P. (2003). Structural characterization of a perdeuterio-methylated exopolysaccharide by NMR spectroscopy: Characterization of the novel exopolysaccharide produced by Lactobacillus delbrueckii subsp. bulgaricus EU23. Carbohydr. Res..

[82] Klare J., Flemming H.-C. (2000). Monitoring of biofouling in papermill process waters. Water Res..

[83] Holm-Hansen O., Booth C.R. (1966). Measurement of adenosine triphosphate in the ocean and its ecological significance. Limnol. Oceanogr..

[84] Hobbie J.E., Daley R.J., Jasper S. (1977). Use of nucleopore filters for counting bacteria by fluorescence microscopy. Appl. Environ. Microbiol..

[85] Van der Kooij D. (1992). Assimilable organic carbon as an indicator of bacterial regrowth. J. Am. Water Work. Assoc..

[86] Van der Kooij D., Hijnen W.A.M., Cornelissen E.R. (2010). Biofouling of Spiral-Wound Membranes in Water Treatment.

[87] Brouwer H., Meesters K., van Groenestijn J. (2006). Biofouling control in reverse osmosis membranes using rapid biofiltration technology. Desalination.

[88] Vrouvenwelder J.S., Kappelhof J.W.N.M., Heiijman S.G.J., Schippers J.C., van der Kooij D. (2003). Tools for fouling diagnosis of NF and RO membranes and assessment of the fouling potential of feed water. Desalination.

[89] Saad M.A. (2004). Early discovery of RO membrane fouling and real-time monitoring of plant performance for optimizing cost of water. Desalination.

[90] Ho B.P., Wu M.W., Zeiher E.H.K., Chattoraj M. (2004). Method of monitoring Biofouling in Membrane Separation Systems. U.S. Patent.

[91] Kujundzic E., Fonseca A.C., Evans E.A., Peterson M., Greenberg A.R., Hermandez M. (2007). Ultrasonic monitoring of early-stage biofilm growth on polymeric surfaces. J. Microbiol. Methods.

[92] Mairal A.P., Greenberg A.R., Krantz W.B. (2000). Investigation of membrane fouling and cleaning using ultrasonic time-domain reflectometry. Desalination.

[93] Li J.X., Sanderson R.D., Chai G.Y. (2006). A focus ultrasonic sensor for in-situ detection of protein fouling on tubular ultrafiltration membranes. Sens. Actuators B.

[94] Lee J., Kim I.S. (2011). Microbial community in seawater reverse osmosis and rapid diagnosis of membrane biofouling. Desalination.

[95] Sung J.H., Chun M.-S., Choi H.J. (2003). On the behaviour of electrokinetic streaming potential during protein filtration with fully and partially retentive nanopores. J. Colloid Interface Sci..

[96] Le-Clech P., Chen V., Fane A.G. (2006). Fouling in membrane bioreactors used in wastewater treatment. J. Membr. Sci..

[97] Teychene B., Loulergue P., Guigui C., Cabassud C. (2011). Development and use of a novel method for inline characterization of fouling layers electrokinetic properties and for fouling monitoring. J. Membr. Sci..

[98] Vrouwenvelder J.S., van Loosdrecht M.C.M., Kruithof J.C. (2011). Early warning of biofouling in spiral wound nanofiltration and reverse osmosis membranes. Desalination.

[99] Chen W.-H., Hsieh Y.-H., Tung K.-L., Li Y.-L., Lai S.-C., Lin N.-J. (2010). An Integrated fouling monitoring technique for a water treatment microfiltration process. Chem. Eng. Technol..

[100] Gorey C., Escobar I.C., Gruden C.L., Cai G. (2010). Development of microbial sensing membranes. Desalination.

[101] Gogate P.R. (2007). Application of cavitational reactors for water disinfection: current status and path forward. J. Environ. Manag..

[102] Saad M.A. (1992). Biofouling prevention in RO polymeric membrane systems. Desalination.

[103] Trägårdh G. (1989). Membrane cleaning. Desalination.

[104] Polanska M., Huysman K., van Keer C. (2005). Investigation of assimilable organic carbon in flemish drinking water. Water Res..

[105] Applegate L.E, Erkenbrcher C.W. (1987). Monitoring and control of biological activity in Permasep seawater RO plants. Desalination.

[106] Joyce E., Phull S.S., Lorimer J.P., Mason T.J. (2003). The development and evaluation of ultrasound for the treatment of bacterial suspensions. A study of frequency, power and sonication time on cultured Bacillus species. Ultrason. Sonochem..

[107] Fagan J., Waite T.D. (1983). Biofouling control with ferrate (IV). Environ. Sci. Technol..

[108] DeLuca S.J., Chao A.C., Smallwood C. (1983). Ames test of ferrate treated water. J. Environ. Eng..

[109] Richardson S.D. (2003). Disinfection by-products and other emerging contaminants in drinking water. Trends Anal. Chem..

[110] Hammes F., Meylan S., Salhi E., Koster O., Egli T., von Gunten U. (2007). Formation of assimilable organic carbon (AOC) and specific natural organic matter (NOM) fractions during ozonation of phytoplankton. Water Res..

[111] Baker J.S., Dudley L.Y. (1998). Biofouling in membrane systems—A review. Desalination.

[112] Hu J.Y., Wang Z.S., Ng W.J., Ong S.L. (1999). The effect of water treatment processes on the biological stability of potable water. Water Res..

[113] Lattemann S. (2010). Development of an Environmental Impact Assessment and Decision Support System for Seawater Desalination Plants. Ph.D. Thesis.

[114] Parrotta M.J., Bekdash F. (1998). UV disinfection of small groundwater supplies. J. Am. Water Work. Assoc..

[115] Lehtola M.J., Miettinen I.T., Vartiainen T., Rantakokko P., Hirvonen A., Martikainen P.J. (2003). Impact of UV disinfection on microbially available phosphorous, organic carbon, and microbial growth in drinking wate. Water Res..

[116] Loge F.J., Darby J.L., Tchobamoglous G. (1996). UV disinfection of wastewater: Probabilistic approach to design. J. Environ. Eng..

[117] Zhou H., Smith D.W. (2002). Advance technologies in water and wastewater treatment. J. Environ. Eng. Sci..

[118] Parker J.A., Darby J.L. (1995). Particle-associated coliform in secondary effluents: shielding from ultra-violet light disinfection. Water Environ. Res..

[119] Harris G.D., Adams V.D., Sorensen D.L., Dupont R.R. (1987). The influence of photoreactivation and water quality on ultraviolet disinfection of secondary municipal wastewater. J. Water Pollut. Control Fed..

[120] Amon R.M.W., Benner R. (1996). Bacterial utilization of different size classes of dissolve organic matter. Limnol. Oceanogr..

[121] Servais P., Laurent P., Randon G. (1995). Comparison of the bacterial dynamics in various French distribution systems. J. Water Supply Res. Technol. Aqua.

[122] Charnock C., Kjønnø O. (2000). Assimilable organic carbon and biodegradable dissolved organic carbon in Norwegian raw and drinking waters. Water Res..

[123] LeChevallier M.W., Becker W.C., Schorr P., Lee R.G. (1992). Evaluating the performance of biologically active rapid filters. J. Am. Water Work. Assoc..

[124] Bradford S.M., Palmer C.J., Olson B.H. (1994). Assimilable organic carbon concentrations in Southern California surface and groundwater. Water Res..

[125] Van der Kooij D., Mc Feters G.A. (1990). Drinking Water Microbiology.

[126] Huck P.M., Fedorak P.M., Anderson W.B. (1991). Formation and removal of assimilable organic carbon during biological treatment. J. Am. Water Work. Assoc..

[127] Van der Kooij D., Hijnen W.A.M., Kruithof J.C. (1989). The effect of ozonation, biological filtration and distribution on the concentration of easily assimilable organic carbon (AOC) in drinking water. Ozone Sci. Eng..

[128] Visvanathan C., Boonthanon N., Sathasivan A., Jegatheesan V. (2002). Pretreatment of seawater for biodegradable organic content removal using membrane bioreactor. Desalination.

[129] van der Hoek J.P., Hofman J.A.M.H., Bonné P.A.C., Nederlof M.M., Vrouwenvelder H.S. (2000). RO treatment: Selection of a pretreatment scheme based on fouling characteristics and operating conditions based on environmental impact. Desalination.

[130] Escobar I.C., Hong S., Randall A.A. (2000). Removal of assimilable organic carbon and biodegradable dissolved organic carbon by reverse osmosis and nanofiltration membranes. J. Membr. Sci..

[131] Vrouwenvelder J.S., Beyer F., Dahmani K., Hasan N., Galjaard G., Kruithof J.C., van Loosdrecht M.C.M. (2010). Phosphate limitation to control biofouling. Water Res..

[132] Morse G.K., Brett S.W., Guy J.A., Lester J.N. (1998). Review: Phosphorus removal and recovery technologies. Sci. Total Environ..

[133] Battistoni P., de Angelis A., Pavan P., Prisciandaro M., Cecchi F. (2001). Phosphorous removal from a real anaerobic supernatant by struvite crystallization. Water Res..

[134] Blaney L.M., Cinar S., SenGupta A.K. (2007). Hybrid anion exchanger for trace phosphate removal from water and wastewater. Water Res..

[135] Jacobs J.F., Hasan M.N., Pai I.K., Hagen W.R., van Loosdrecht M.C. (2010). Development of a bionanotechnological phosphate removal system with thermostable ferritin. Biotechnol. Bioeng..

[136] Eliassen R., Tchobanoglous G. (1969). Removal of nitrogen and phosphorus from wastewater. Environ. Sci. Technol..

[137] Fytianos K., Voudrias E., Raikos N. (1998). Modelling of phosphorous removal from aqueous and wastewater samples using ferric iron. Environ. Pollut..

[138] Vasudevan S., Sozhan G., Ravichandran S., Jayaraj J., Lakshmi J., Sheela S.M. (2008). Studies on the removal of phosphate from drinking water by electrocoagulation process. Ind. Eng. Chem. Res..

[139] Chen G. (2004). Electrochemical technologies in wastewater treatment. Sep. Purif. Technol..

[140] Karaca S., Gǔrses A., Ejer M., Açikildiz M. (2006). Adsorption removal of phosphate from aqueous solutions using raw and calcined dolomite. J. Hazard. Mater..

[141] Stensel H.D. (1991). Principal of Biological Phosphorus Removal: Phosphorus and Nitrogen Removal from Municipal Wastewater—Principles and Practices.

[142] Gonzalez J.E., Keshavan N.D. (2006). Messing with bacterial quorum sensing. Microbiol. Mol. Biol. Rev..

[143] Xiong Y., Liu Y. (2010). Biological control of microbial attachment: A promising alternative for mitigating membrane biofouling. Appl. Microbiol. Biotechnol..

[144] Xavier K.B., Bassler B.L. (2003). LuxS quorum sensing: More than just a numbers game. Curr. Opin. Microbiol..

[145] Richards J.J., Melander C. (2009). Controlling of bacterial biofilms. ChemBioChem.

[146] Kim S.J., Lee S.Y., Hong S.K., Oh Y.S., Seoul M.J., Kweon J.H., Kim T.H. (2009). Biofouling of reverse osmosis membranes: Microbial quorum sensing and fouling propensity. Desalination.

[147] Ponnusamy K., Paul D., Kim Y.S., Kweon J.H. (2010). 2(5H)-Furanone: A prospective strategy for biofouling-control in membrane biofilm bacteria by quorum-sensing inhibition. Braz. J. Microbiol..

[148] Kappachery S., Paul D., Yoon J., Kweon J.H. (2010). Vanillin, a potential agent to prevent biofouling of reverse osmosis membranes. Biofouling.

[149] Yeon K.-M., Cheong W.-S., Oh H.-S., Lee W.-N., Huang B.-K., Lee C.-H., Beyenal H., Lewandowski Z. (2009). Quorum sensing: a new biofouling control paradigm in a membrane bioreactor for advanced wastewater treatment. Environ. Sci. Technol..

[150] Mc Grath S. (2007). Bacteriophage: Genetics and Molecular Biology.

[151] Goldman G., Starosvetsky J., Armon R. (2009). Inhibition of biofilm on UF membrane by use of specific bacteriophages. J. Membr. Sci..

[152] Araki M. (1986). Advanced slime control process with bacteriophage. Kogyo Yosui.

[153] Brockhurst M.A., Buckling A., Rainey P.B. (2006). Spatial heterogeneity and the stability of host-parasite coexistence. J. Evol. Biol..

[154] Berraud N., Storey M.V., Moore Z.P., Webb J.S., Rice S.A., Kjelleberg S. (2009). Nitric-oxide- meditated dispersal in single- and multi-species biofilms of clinically and industrially relevant micro-organisms. Microb. Biotechnol..

[155] Charville G.W., Hetric E.M., Geer C.B., Schoenfisch M.H. (2008). Reduced bacterial adhesion to fibrinogen-coated substrates via nitric oxide release. Biomaterials.

[156] Cai T.B., Wang P.G., Holder A.A., Wang P.G., Cai T.B., Taniguchi N. (2005). NO and NO donors. Nitric oxide donors.

[157] Mollee T.R., Anissimov Y.G., Roberts M.S. (2006). Periodic electric field enhanced transport through membranes. J. Membr. Sci..

[158] Brunner G., Okoro E. (1989). Reduction of membrane fouling by means of electric field during ultrafiltration of proteins solutions. Ber. Bunsenges. Phys. Chem..

[159] Jagannadh S.N., Muralidhara H.S. (1996). Electrokinetic methods to control membrane fouling. Ind. Eng. Chem. Res..

[160] Brors A. (1992). Untersuchungen zum Einfluβ von elektrischen Feldern bei der Querstromfiltration von biologischen Suspensionen. Fortschritt–Berichte VDI Reihe 3, Nr. 284.

[161] Zumbusch P.V., Kulcke W., Brunner G. (1998). Use of alternating electrical fields as anti-fouling strategy in ultrafiltration of biological suspensions—Introduction of a new experimental procedure for cross flow filtration. J. Membr. Sci..

[162] Volk C., Bell K., Ibrahim E., Verges D., Amy G., Le Chevallier M. (2000). Impact of enhanced and optimized coagulation on removal of organic matter and its biodegradable fraction in drinking water. Water Res..

[163] Kim S.-H., Lee H.-I., Yoon C.-H. (2003). Evaluation of flocculation performance using floc characteristics. J. Korean Soc. Water Wastewater.

[164] Shon H.K., Vigneswaran S., Kim I.S., Cho J., Ngo H.H. (2004). Effect of pretreatment on the fouling of membranes: Application in biologically treated sewage effluent. J. Membr. Sci..

[165] Song K.G., Kim Y., Ahn K.H. (2008). Effect of coagulant addition on membrane fouling and nutrient removal in a submerged membrane bioreactor. Desalination.

[166] Tran T.T., Shafiquzzaman M., Nakajima J. (2010). Control of membrane fouling by coagulant and coagulant aid addition in membrane bioreactor systems. J. Water Environ. Technol..

[167] Wu J., Chen F., Huang X., Geng W., Wen X. (2006). Using inorganic coagulants to control membrane biofouling in a submerged membrane bioreactor. Desalination.

[168] Henderson R., Parsons S.A., Jefferson B. (2008). The impact of algal properties and pre-oxidation on solid-liquid separation of algae. Water Res..

[169] Bernhardt H., Clasen J. (1991). Flocculation of micro-organisms. J. Water Supply Res. Technol. Aqua.

[170] Pieterse A.J.H., Cloot A. (1997). Algal cells and coagulation, flocculation and sedimentation processes. Water Sci. Technol..

[171] Pivokonsky M., Kloucek O., Pivokonska L. (2006). Evaluation of the production, composition and aluminum and iron complexation of algogenic organic matter. Water Res..

[172] Bernhardt H., Hoyer O., Schell H., Lusse B. (1995). Reaction mechanisms involved in the influence of algogenic matter on flocculation. Z. Wasser Abwasser Forsch..

[173] Wilf M., Alt S. (2000). Application of low fouling RO membrane elements for reclamation of municipal wastewater. Desalination.

[174] Gabelich C.J., Yun T.I., Coffey B.M., Suffet I.H. (2002). Effects of aluminum sulphate and ferric chloride coagulant residual on polyamide membrane performance. Desalination.

[175] Bereschenko L.A., Prummel H., Euverink G.J.W., Stams A.J.M., van Loosdrecht M.C.M. (2011). Effect of conventional chemical treatment on the microbial population in a biofouling layer of reverse osmosis systems. Water Res..

[176] Kim S.L., Chen J.P., Ting Y.P. (2002). Study on feed pretreatment for membrane filtration of secondary effluent. Sep. Purif. Technol..

[177] Silvestry-Rodriguez N., Bright K.R., Slack D.C., Uhlmann D.R., Gerba C.P. (2008). Silver as a residual disinfectant to prevent biofilm formation in water distribution systems. Appl. Environ. Microbiol..

[178] Lok C.N., Ho C.M., Chen R., He Q.Y., Yu W.Y., Sun H.Z., Tam K.H., Chiu J.F., Che C.M. (2006). Proteomic analysis of the mode of antibacterial action of silver nanoparticles. Proteome Res..

[179] Dror-Ehre A., Adin A., Markovick G., Mamane H. (2010). Control biofilm formation in water using molecularly capped silver nanoparticles. Water Res..

[180] Hilal N., Al-Khatib L., Atkin B.P., Kochkodan V., Potapchenko N. (2003). Photochemical modification of membrane surfaces for biofouling reducing: A nano-scale study using AFM. Desalination.

[181] Kim J.-H., Lee K.-H. (1998). Effect of PEG additive on membrane formation by phase inversion. J. Membr. Sci..

[182] Van der Bruggen B. (2009). Chemical modification of polyethersulfone nanofiltration membranes: a review. J. Appl. Polym. Sci..

[183] Khulbe K.C., Feng C., Matsuura T. (2010). The art of surface modification of synthetic polymeric membrane. J. Appl. Polym. Sci..

[184] Bae T.-H., Tak T.-M. (2005). Effects of TiO_2_ nanoparticles on fouling mitigation of ultrafiltration membranes for activated sludge filtration. J. Membr. Sci..

[185] Khayet M., Villaluenga J.P.G., Valentin J.L., Lopez-Manchado M.A., Mengual J.I., Seoane B. (2005). Filled poly(2,6-dimethyl-1,4-phenylene oxide) dense membranes by silica and silane modified silica nanoparticles: characterization and application in pervaporation. Polymer.

[186] Yan L., Li Y.S., Xiang C.B. (2005). Preparation of poly(vinylidene fluoride) (PDVF) ultrafiltration membrane modified by nano-sized alumina (Al_2_O_3_) and its antifouling research. Polymer.

[187] Bottino A., Capannelli G., Comite A. (2002). Preparation and characterization of novel porous PVDF-ZrO2 composite membranes. Desalination.

[188] Lin D.-J., Chang C.-L., Huang F.-M., Cheng L.-P. (2003). Effect of salt additive on the formation of microporous poly(vinylidene fluoride) membranes by phase inversion from LiClO_4_/water/DMF/PVDF system. Polymer.

[189] Zhang X., Du A.J., Lee P., Sun D.D., Leckie J.O. (2008). TiO_2_ nanowire membrane for concurrent filtration and photocatalytic oxidation of humic acid in water. J. Membr. Sci..

[190] Tashiro T. (2001). Antibacterial and bacterium adsorbing macromolecules. Macromol. Mater. Eng..

[191] Desai N.P., Hossainy S.F.A., Hubbell J.A. (1992). Surface-immobilized polyethylene oxide for bacterial repellence. Biomaterials.

[192] Borkow G., Gabbay J. (2005). Copper as a biocidal tool. Curr. Med. Chem..

[193] Feng Q.L., Wu J., Chen G.Q., Cui F.Z., Kim T.N., Kim J.O. (2000). A mechanistic study of antibacterial effect of silver ion on Escherichia coli and Staphylococcus aureus. J. Biomed. Mater. Res. A.

[194] Liu C.X., Zhang D.R., He Y., Zhao X.S., Bai R. (2010). Modification of membrane surface for anti-biofouling performance: Effect of anti-adhesion and anti-bacteria approaches. J. Membr. Sci..

[195] Dror-Ehre A., Mamane H., Belenkova T., Markovich G., Adin A. (2009). Silver nanoparticles—*E. coli* interaction in water and effect on *E. coli* survival. J. Colloid Interface Sci..

[196] Zodrow K., Brunet L., Mahendra S., Li D., Zhang A., Li Q., Alvarez P.J.J. (2009). Polysulfone ultrafiltration membranes impregnated with silver nanoparticles show improved biofouling resistance and virus removal. Water Res..

[197] Chae S.-R., Wang S., Hendren Z.D., Weisner M.R., Wantanabe Y., Gunsch C.K. (2009). Effect of fullerene nanoparticles on Escherichia coli K12 respiratory activity in aqueous suspension and potential use for membrane biofouling control. J. Membr. Sci..

[198] Liu P.-S., Chen Q., Wu S.-S., Shen J., Lin S.-C. (2010). Surface modification of cellulose membranes with zwitterionic polymers for resistance to protein adsorption and platelet adhesion. J. Membr. Sci..

[199] Zhang Z., Cheng S., Chang Y., Jiang S. (2006). Surface grafted sulfobetaine polymers via atom transfer radical polymerization as superlow fouling coatings. J. Phys. Chem. B.

[200] Ye S.H., Watanabe Y., Iwasaki K., Ishihara K. (2002). Novel cellulose acetate membrane blended with phospholipid polymer for hemocompatible filtration system. J. Membr. Sci..

[201] Wang D., Williams C.G., Li Q., Sharma B., Elisseef J.H. (2003). Synthesis and characterization of a novel degradable phosphate containing hydrogel. Biomaterials.

[202] Ishihara K., Ueda T., Nakabayashi N. (1992). Preparation of 2-methacryloyloxyethyl phosphorylcholine copolymers with alkyl methacrylates and their blood compatibility. Polym. J..

[203] Shi Q., Su W., Zhao W., Li C., Hu Z., Jiang S., Zhu S. (2008). Zwitterionic polyethersulfone ultrafiltration membrane with superior antifouling property. J. Membr. Sci..

[204] Chiang Y.-C., Chang Y., Higuchi A., Chen W.-Y., Ruaan R.-C. (2009). Sulfobetaine-grafted poly(vinylidene fluoride) ultrafiltration membranes exhibit excellent antifouling property. J. Membr. Sci..

[205] Zhao Y.-H., Wee K.-H., Bai R. (2010). Highly hydrophilic and low protein-fouling propylene membrane prepared by surface modification with sulfobetaine-based Zwitterionic polymer through a combine surface polymerization method. J. Membr. Sci..

[206] Zhao Y.-H., Zhu X.-Y., Wee K.-H., Bai R. (2010). Achieving high effective non-biofouling performance for polypropylene membranes modified by UV-induced surface graft polymerization of two oppositely charged monomers. J. Phys. Chem. B.

[207] Ostuni E., Chapman R.G., Holmlin R.K., Takayama S., Whitesides G.M. (2001). A survey of structure-property relationships of surface that resist the adsorption of protein. Langmuir.

[208] Susanto H., Ulbricht M. (2007). Photografted thin polymer hydrogel layer on PES ultrafiltration membranes: Characterization, stability, and influence on separation performance. Langmuir.

[209] Chen V., Fan A.G., Fell C.J.D. (1992). The use of anionic surfactants for reducing fouling of ultrafiltration membranes: Their effects and optimization. J. Membr. Sci..

[210] Brink L.E.S., Romijn D.J. (1990). Reducing the protein fouling of polysulfone surfaces and polysulfone ultrafiltration membranes: Optimization of the type of pre-adsorbed layer. Desalination.

[211] Watanabe N., Shirakawa T., Iwahasi M., Seimiya T. (1986). Effect of surface charge on absorption of biovine serum albumin as studied by ellipsometry II. Interaction of protein molecules with an anionic monolayer as studied by ellipsometry, radiotracer and surface tension measurements. Colloid Polym. Sci..

[212] Mansouri J., Harrisson S., Chen V. (2010). Strategies for controlling biofouling in membrane filtration systems: Challenges and opportunities. J. Mater. Chem..

[213] Schwinge J., Neal P.R., Wiley D.E., Fletcher D.F., Fane A.G. (2004). Spiral wound modules and spacers: Review and analysis. J. Membr. Sci..

[214] Williams C. (2000). Membrane fouling and alternative techniques for its alleviation. Membr. Technol..

[215] Vrouwenvelder J.S. (2009). Biofouling of Spiral Wound Membrane System. Ph.D. Thesis.

[216] Liao B.Q., Bagley D.M., Kramer H.E., Leppard G.G., Liss S.N. (2004). A review of biofouling and its control in membrane separation bioreactors. Water Environ. Res..

[217] Flemming H.-C., Flemming H.-C., Geesey G.G. (1991). Biofouling and Biocorrosion in Industrial Water Systems.

[218] Madaeni S.S., Mohamamdi T., Moghadam M.K. (2001). Chemical cleaning of reverse osmosis membranes. Desalination.

[219] Hilal N., Ogunbiyi O.O., Miles N.J., Nigmatullin R. (2005). Methods employed for control of fouling in MF and UF membranes: A comprehensive review. Sep. Sci. Technol..

[220] Pearce G. (2007). Introduction to membrane: Fouling control. Filtr. Sep..

[221] Lin J.C.-T., Lee D.-J., Huang C. (2010). Membrane fouling mitigation: Membrane cleaning. Sep. Sci. Technol..

[222] An Y., Wu B., Wong F.S., Yang F. (2010). Post-treatment of upflow anaerobic sludge blanket effluent by combining the membrane filtration process: Fouling control by intermittent permeation and air sparging. Water Environ. J..

[223] Cornelissen E.R., Vrouwenvelder J.S., Heijman S.G.J., Viallefont X.D., van der Kooij D., Wessels L.P. (2007). Periodic air/water cleaning for control of biofouling in spiral wound membrane elements. J. Membr. Sci..

[224] Ebrahim S. (1994). Cleaning and regeneration of membrane in desalination and wastewater applications: State of the art. Desalination.

[225] Lörincz A. (2004). Ultrasonic cellular disruption of yeast in water-based suspensions. Biosyst. Eng..

[226] Lamminen M.O., Walker H.W., Weavers L.K. (2004). Mechanisms and factors influencing the ultrasonic cleaning of particle-fouled ceramic membranes. J. Membr. Sci..

[227] Masselin I., Chasseray X., Laurence D.B., Lain J.-M., Syzaret P.-Y., Lemordant D. (2001). Effect of sonication on polymeric membranes. J. Membr. Sci..

[228] Lu J.-Y., Du X., Lipscomb G. Cleaning membranes with focused ultrasound beams for drinking water treatment. Proceedings of 2009 IEEE International Ultrasonics Symposium.

[229] Kyllönen H.M., Pirkonen P., Nyström M. (2005). Membrane filtration enhanced by ultrasound: A review. Desalination.

[230] Lamminen M.O. (2005). Ultrasonic Cleaning of Latex Particle Fouled Membranes. Ph.D. Thesis.

[231] Tarazaga C.C., Campderrós M.E., Padilla A.P. (2006). Physical cleaning by means of electrical field in the ultrafiltration of a biological solution. J. Membr. Sci..

[232] Agarwal A., Xu H., Ng. W.J., Liu Y. (2012). Biofilm detachment by self-collapsing air microbubbles: a potential chemical-free cleaning technology for membrane biofouling. J. Mater. Chem..

[233] Zondervan E., Roffel B. (2007). Evaluation of different cleaning agents used for cleaning ultra-filtration membranes fouled by surface water. J. Membr. Sci..

[234] Liu C., Caothien S., Hayes J., Caothuy T., Otoyo T., Ogawa T. Membrane chemical cleaning: From art to science. http://www.pall.com/water_8158.asp.

[235] Thurman E.M. (1985). Organic Chemistry of Natural Waters.

[236] Rosen M.J. (1989). Surfactant and Interfacial Phenomena.

[237] Ang W.S., Lee S., Elimelech M. (2006). Chemical and physical aspects of cleaning of organic fouled reverses osmosis membrane. J. Membr. Sci..

[238] Rosenberg M., Doyle R.J. (1990). Microbial Cell Surface Hydrophobicity.

[239] Herzberg M., Elimelech M. (2007). Biofouling of reverse osmosis membranes: Role of bio-film-enhanced osmotic pressure. J. Membr. Sci..

[240] Allie Z., Jacobs E.P., Maartens A., Swart P. (2003). Enzymatic cleaning of ultrafiltration fouled by abattoir effluent. J. Membr. Sci..

[241] Loiselle M., Anderson K.W. (2003). The use of cellulose in inhibiting biofilm formation from organisms commonly found on medical implants. Biofouling.

[242] Leroy C., Delbarre-Ladrat C., Ghillebaert F., Compere C., Combes D. (2008). Influence of subtilising on the adhesion of a marine bacterium which produces mainly proteins as extracellular polymers. J. Appl. Microbiol..

[243] Brady D., Jordan J. (2009). Advances in enzyme immobilisation. Biotechnol. Lett..

[244] Mikkelsen L.H., Keiding K. (2002). Physico-chemical characteristics of full scale sewage sludge with implications to dewatering. Water Res..

[245] Xu H., Liu Y. (2011). Control and cleaning membrane biofouling by energy uncoupling and cellular communication. Environ. Sci. Technol..

[246] Kierek-Pearson K., Karatan E. (2005). Biofilm development in bacteria. Adv. Appl. Microbiol..

[247] Flemming H.C. (2002). Biofouling in water systems—cases, causes, countermeasures. Appl. Environ. Biotechnol..

[248] Madaeni S.S., Mansourpanah Y. (2004). Chemical cleaning of reverse osmosis membranes fouled by whey. Desalination.

[249] Chen J.P., Kim S.L., Ting Y.P. (2003). Optimization of membrane physical and chemical cleaning by a statistically designed approach. J. Membr. Sci..

[250] Al-Amoudi A., Lovitt R.W. (2007). Fouling strategies and cleaning system of NF membranes and factors affecting cleaning efficiency. J. Membr. Sci..

